# Molecular and neural control of social hierarchy by forebrain-thalamocortical circuit

**DOI:** 10.1016/j.cell.2025.07.024

**Published:** 2025-08-11

**Authors:** Adam C. Nelson, Vikrant Kapoor, Eric Vaughn, Jeshurun A. Gnanasegaram, Nimrod D. Rubinstein, Mustafa Talay, Venkatesh N Murthy, Catherine Dulac

**Affiliations:** 1 Howard Hughes Medical Institute, Department of Molecular and Cellular Biology, Center for Brain Science, Harvard University, Cambridge, MA, USA.; 2 Present address: Department of Zoology and Physiology, University of Wyoming, Laramie, WY, USA; 3 Current address: Calico Life Sciences LLC, 1170 Veterans Blvd., South San Francisco, USA; 4 Department of Molecular and Cellular Biology, Center for Brain Science, Harvard University, Cambridge, MA, USA.

## Abstract

Many animal groups are organized hierarchically, which generates behavioral states that facilitate social interactions. Although generally stable, social status can change, underscoring the plasticity of underlying neural circuits. We examined competition among unfamiliar male mice and uncovered how the molecular and biophysical characteristics of a forebrain-thalamocortical circuit affect hierarchy. We identify the mediodorsal thalamus (MDT) as a hub receiving inputs from orbitofrontal cortex and basal forebrain and projecting to caudal anterior cingulate cortex (cACC) to regulate competitive performance. This circuit becomes potentiated or depressed in high- and low-rank males, respectively, in part through altered expression of the voltage-gated ion channel *Trpm3* and synaptic plasticity. In high-rank mice, MDT projections drive inhibition of cACC pyramidal cells, promoting winning, in a pattern strikingly opposite to dorsomedial prefrontal cortex, where winners display increased pyramidal cell activity. Our data suggests a model in which hierarchy modulation relies on coordinated remodeling of multiple forebrain-thalamocortical circuits.

## In Brief

Social hierarchy in unfamiliar male mice is regulated by the mediodorsal thalamus acting as a hub whose altered expression of the voltage-gated ion channel Trpm3 and synaptic plasticity drive inhibition of cACC pyramidal cells and increased activity of dmPFC pyramidal cells in high-ranking mice.

## Graphical Abstract

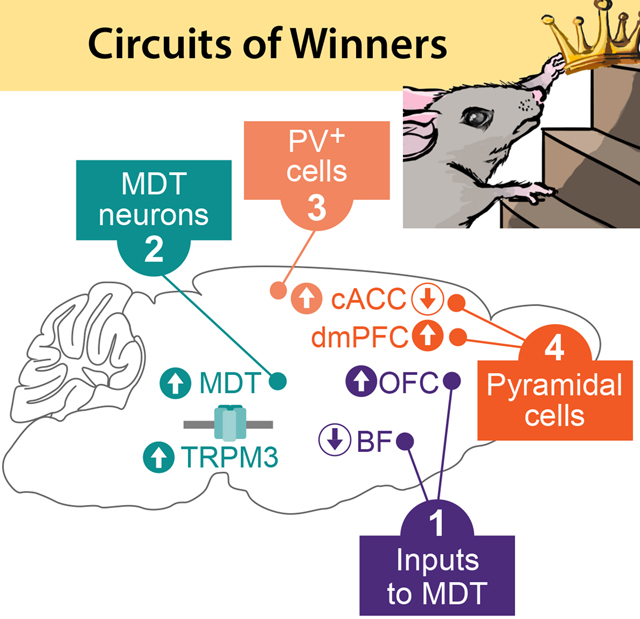

## INTRODUCTION

Understanding the molecular and circuit mechanisms underlying flexible behavioral states remains a key challenge in neuroscience and neuropsychiatry^1,2^. Social status, a behavioral state conveyed through dominant or submissive displays, is aimed at reducing interpersonal conflicts^3^. Although generally stable, social status can change in response to physiological and environmental conditions.

An individual’s social status is typically determined through pairwise interactions with group members. Social hierarchy forms when these interactions stabilize in competitive contexts ^4,5^. Social hierarchy behavior emerges early in life and becomes more structured into adulthood, with males and females exhibiting sex-specific strategies for establishing rank ^6 7,8^. Within groups, the social status of each member is a key determinant of individual differences in behavior and mental health^4,7,9^.

Hierarchy is regulated across multiple scales of biological organization, from the group (social signals), to the cellular (neural activity and connectivity) and molecular (signaling pathways and hormones) levels^10–12^. Human imaging studies during competition show that opponent dominance status is reflected in rostral medial prefrontal cortex (PFC) activity patterns^13^: viewing superior individuals engages the dorsolateral PFC ^12^, while knowledge of own’s status involves the anterior cingulate area (ACC) ^14^.

Recent efforts have aimed at understanding how social hierarchy is regulated by neural circuits^15–17^. House mice form hierarchies in wild and laboratory settings, providing an attractive model to study underlying molecular and neural mechanisms ^11,15,18^. Tube test assays measuring win-or-lose pairwise interactions have shown that, in cohoused lab mice, a subset of males spontaneously display stable hierarchies ^15,19,20^. Investigation of these hierarchies revealed that activation of a local cortical disinhibitory circuit in the dorsomedial prefrontal cortex (dmPFC), and of upstream thalamic inputs, increases social rank ^19–21^.

Here, we sought to gain a broad perspective on brain regions, cell-types, and neuronal properties associated with social hierarchy. We developed a behavioral paradigm that assesses the establishment and maintenance of hierarchy among unfamiliar male mice, and used brain-wide activity mapping, as well as molecular, genetic, and biophysical tools for circuit identification and manipulation. We identified a multisynaptic circuit regulating social hierarchy that comprises the mediodorsal thalamus (MDT), inputs from orbitofrontal cortex (OFC) and basal forebrain (BF), and projections to caudal anterior cingulate area (cACC). Enhanced MDT activity in high-rank males involves increased TRPM3 activity, potentiated excitatory OFC→MDT connectivity, and reduced inhibitory BF→MDT connectivity. MDT projections to cACC drive feedforward inhibition, with high-rank males displaying reduced cACC activity compared to low-rank males—opposite to the previously described pattern in dmPFC^20,21^. Our data suggests an updated model in which responses to conspecific stimuli during competitive encounters rely on coordinated but opposite feedforward inhibition and feedforward excitation in forebrain→MDT→cACC and MDT→dmPFC circuits, respectively. This study provides mechanistic insights into the coordinated and multiscale modulation of multi-synaptic forebrain-thalamocortical circuits underlying competitive behavior across genes, synapses and circuits.

## RESULTS

### Emergence of social hierarchy in mice

**Figure 1.**
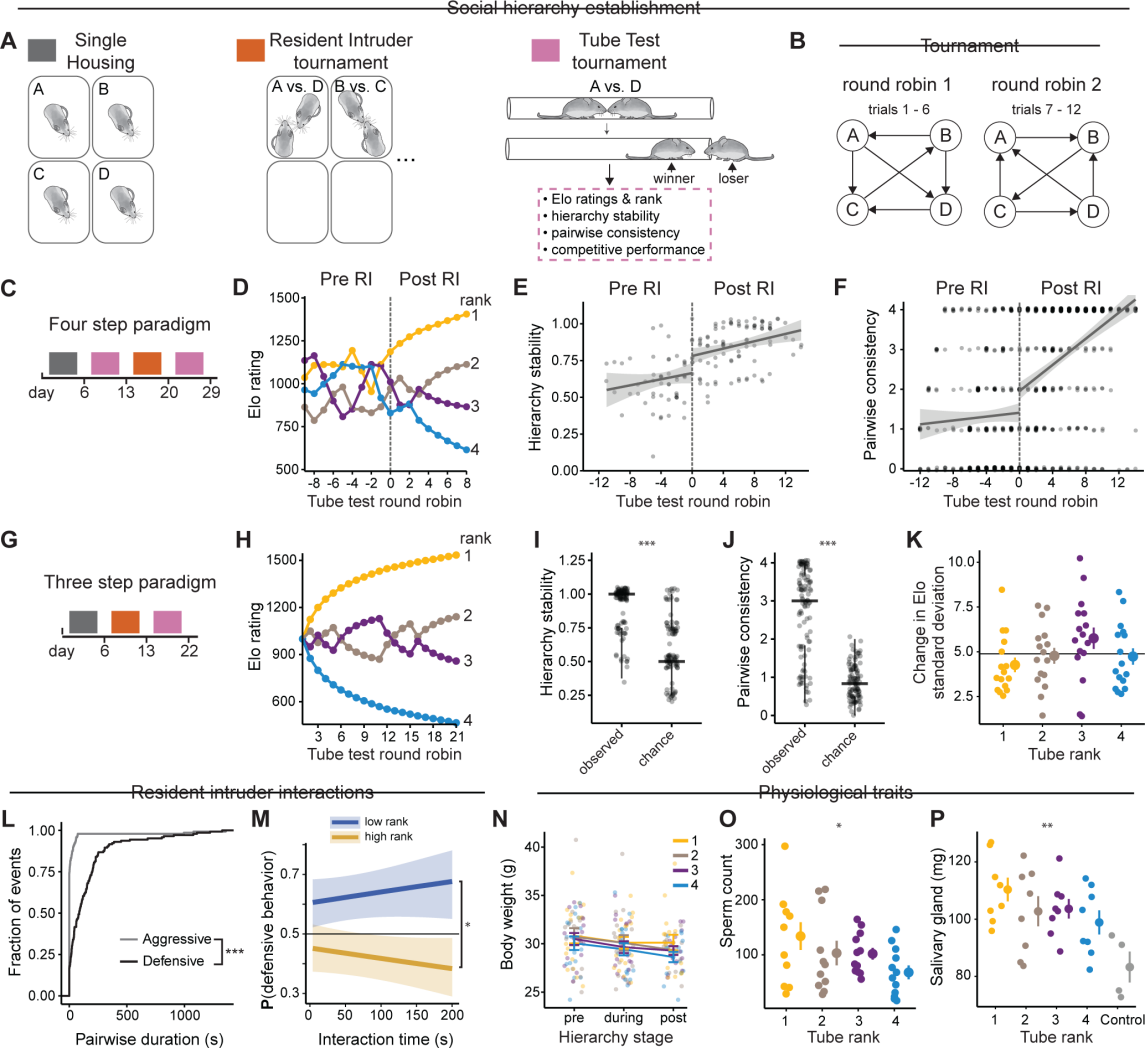
Determinants of hierarchy formation **(A - B)** Behavioral assays and tournament structure. **(A)** Single housing (SH); resident-intruder (RI) tournament; tube test (TT) tournament. **(B)** Round robin tournament structure. **(C)** Four-step paradigm. **(D)** Tube test Elo ratings before/after RI tournament. **(E-F)** Hierarchy stability **(E)** and pairwise consistency **(F)** in tube test tournaments before/after resident-intruder tournament. Dots: mean stability per group in (E) and mean consistency per pairwise interaction (F). Lines: linear model ± s.e.m. **(G)** Three-step paradigm. **(H)** Elo ratings and ordinal ranks in group of four. **(I-J)** Tube test hierarchy stability **(I)** and pairwise consistency **(J)** of observed groups compared to random outcomes. Box plots: median and first and third quartiles (N = 12 groups of 4 mice). **(K)** Standard deviation in delta Elo scores (N = 16 groups of 4 mice, effect of rank: p<0.001). Horizontal line: mean standard deviation. Large dots: mean ±s.e.m. **(L)** Cumulative distribution function plot of aggressive vs. defensive events in resident-intruder interactions (N = 12 groups of 4 mice). **(M)** Probability of defensive behavior by high and low ranks. **(N-P)** Physiological correlates of social rank. Body weight throughout hierarchy formation (N = 18 groups of 4 mice) **(N)**. Relative sperm count **(O)** and salivary gland mass **(P)** *vs*. final tube rank (N = 4 groups of 4 mice plus 2 control mice). LMER: panels I, J, K, N and P. Binomial logistic regression: panel M. Data are mean ± s.e.m. * p<0.05. ** p<0.01. ***p<0.001.

We sought to uncover neural mechanisms underlying the emergence and maintenance of stable hierarchy in naïve cohorts of individuals without prior social interaction. Cohorts of four single-housed adult C57BL/6J males (~ eight weeks old) were introduced to each other through pairwise interactions in two distinct round robin assays: resident-intruder and tube tests, each performed daily over approximately one week (Fig. 1A & B). Resident-intruder assays measure agonistic behavior during intrusions into the home cage ^22^, and tube tests elicit pairwise head-to-head interactions where the first mouse to exit the tube is deemed the loser ^19^.

Hierarchical relationships in tube tests were scored with Elo ratings, which dynamically update based on performance relative to expectations from previous contests^23^, with unexpected outcomes causing larger point changes. Probabilistic models like Elo offer advantages over ordinal ranking by accommodating ties, quantifying dominance magnitude, and enabling parametric statistical analysis ^24^. Where appropriate, we used Linear Mixed Effect Models (LMERs) to identify correlates of social rank while controlling for between-group variation and batch effects. Additionally, we monitored intra-group hierarchy stability (i.e., frequency of rank changes) and pairwise consistency (i.e., the repeatability of each pairwise interaction).

Groups of four mice assayed in tube tests tournaments showed low hierarchy stability and pairwise consistency prior to any mutual social contact through resident-intruder interactions (Fig. 1C–F, Pre RI). However, exposure to territorial interactions through resident-intruder round robin assays promoted stable hierarchy formation in subsequent tube tests (Fig. 1C–F, Post RI). Next, we evaluated the establishment of hierarchy in a three-step paradigm: (1) single housing, (2) resident-intruder tournaments, and (3) tube test tournaments, each lasting approximately one week (Fig. 1G). This approach produced clear social ranks by Elo rating (Fig. 1H) with hierarchies more stable (Fig. 1I) and showing greater pairwise consistency (Fig. 1J) than by chance. Rank-dependent variance analysis revealed rank-1 mice had below-average variance while rank-3 mice showed above-average variance (Fig, 1K), indicating that high-rank mice maintain position more consistently than low-rank mice.

Similarly, groups of three mice subjected to this three-step paradigm showed high levels of hierarchy stability and pairwise consistency with rank-1 mice showing lower variance in competitive performance compared to other ranks (SOM Fig. 1A–D). We used this three-step hierarchy paradigm (Fig. 1G) applied to groups of three or four initially unfamiliar mice in all subsequent experiments and manipulations.

We next assessed how resident-intruder interactions predicted tube test outcomes. Defensive (e.g., avoidance) behaviors were observed in every trial and more frequently than aggressive (i.e., attack) behaviors (Fig. 1L). Logistic regression showed that mice achieving higher ranks (rank-1 and rank-2) displayed less defensive behavior during resident-intruder tests than lower-ranked mice (rank-3 and rank-4) (Fig. 1M). Relative duration of defensive behavior (calculated as A/(A+B) for mouse A versus mouse B) better predicted tube rank than absolute defensive duration (SOM Fig. 1E–G), while aggressive behavior probability showed no rank correlation (SOM Fig. 1H). These results suggest that mice dynamically modulate defensive responses based on competitor status and that increased defensive behavior in low-rank, rather than aggressive behavior in high-rank, characterizes hierarchy formation among unfamiliar males.

### Physiological correlates of social rank

We next investigated physiological traits and social cues associated with hierarchy in groups of four. Although mice lost weight during hierarchy formation, social rank showed no correlation with weight at any stage (Fig. 1N). Testosterone has been associated with aggression but evidence is contradictory ^25,26^, prompting us instead to measure androgen-dependent traits ^27,28^ as readouts of testosterone levels. We found that sperm count and salivary gland mass were higher in rank-1 than rank-4 males (Fig. 1O,P).

To further investigate the role of chemosensory cues, we measured competitive performance when saliva, urine, or both were swabbed onto rank-1 and rank-4 mice. While saliva did not affect tube test interactions, urine from rank-1 males transiently increased competitive performance of rank-4 males in 66% of trials (SOM Fig. 1I, J). Transferring urine from rank-4 to rank-1 had no effect. To investigate other rank-dependent cues, we measured ultrasonic vocalizations and found no emission during tube tests (SOM Fig. 1K). Thus, social rank is associated with androgen-dependent traits and, as shown previously, urinary cues signal high rank^29^.

Together, we established a behavioral paradigm where hierarchy emerges among separately housed mice, revealing that high rank corresponds with decreased defensive behavior—unlike cohoused males where it correlates with increased aggression^19^.

### Neural activity patterns in tube test tournaments

**Figure 2.**
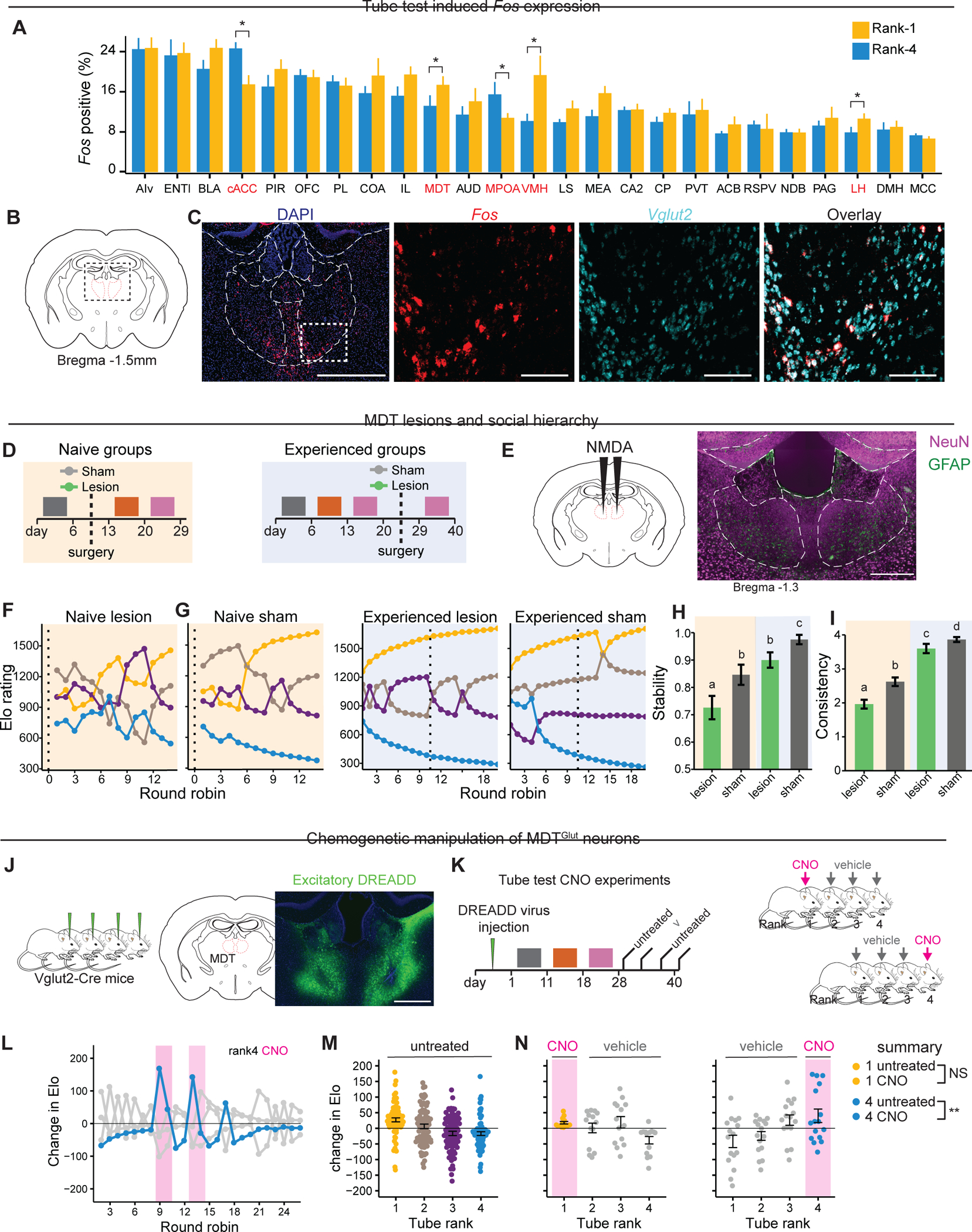
Social rank-dependent brain activity and requirement of MDT in hierarchy emergence. **(A)** *Fos* expression in 25 brain regions from rank-1 and rank-4 males. N = 4 groups. **(B)** MDT schematic. **(C)** DAPI+, *Fos*+, *Vglut2*+ cells and *Fos*-*Vglut2* overlap in MDT. **(D)** Lesion timeline. **(E)** NMDA injections (left) with NeuN and GFAP staining of MDT (right). **(F-I)** Lesion effects on hierarchy dynamics in naïve **(F)** and experienced **(G)** groups. Mean hierarchy stability **(H)** and consistency **(I)** during tube test tournaments in naïve and experienced groups. Wilcoxon rank sum test, bars not connected by same letter are significantly different (p<0.05). **(J)** Strategy for DREADD expression in Vglut2-Cre mice. **(K)** Timeline. **(L-N)** Effect MDT^Glut^ excitation on competitive performance. N = 4 groups. **(L)** Example CNO delivery (magenta rectangle) to rank-4 male (blue trace). **(M)** Performance *vs*. final tube rank in untreated mice. **(N)** Effect of CNO delivery to rank-1 (left) or rank-4 (right). * p<0.05,. LMER with Tukey post hoc test (** p<0.01). Data are mean ± s.e.m.. Meaning of abbreviations in A are in corresponding SOM Fig.2A legend.

To identify brain areas associated with social hierarchy, we compared the number of cells expressing the immediate early gene *Fos* in rank-1 *vs.* rank-4 males following tube test tournaments (Fig. 2A), as well as in individually housed controls passed through the tube without social interaction (SOM Fig. 2A). Using fluorescent in situ hybridization (FISH) and an automated image analysis pipeline, we analyzed 25 brain regions associated with primate social hierarchy ^30^, or seen as activated (i.e., changes in *Fos* expression) in pilot experiments.

Using LMER models to control for group effects and batch effects in histology processing, we found similar *Fos* expression patterns in rank-1 and rank-4 mice, both in brain areas expressing *Fos* and in number of *Fos*-positive cells. However, five regions showed significant rank-dependent differences (Fig. 2A). Compared to rank-4, rank-1 males showed increased activity in the ventromedial hypothalamus, lateral habenula, and the mediodorsal thalamus (MDT) (Fig. 2A–C), consistent with previous findings that MDT→dmPFC activation affects social rank in cohoused mice ^20^. By contrast, compared to rank-1, rank-4 showed increased activity in the medial preoptic area (MPOA) and the anterior cingulate cortex (ACC), including the region proximal to the genu of the corpus collosum, also known as caudal ACC (cACC).

To explore potential functional connectivity, we evaluated rank-dependent pairwise correlations in *Fos* expression across the 25 brain regions (SOM Fig. 2B). Nine correlations differed significantly between rank-1 and rank-4, all involving either MDT (4/9) or cACC (5/9) (SOM Fig. 2B, right). Social rank affected MDT *Fos* correlations with three cortical areas (prelimbic area (PL), orbitofrontal cortex (OFC), and cACC) as well as with the hippocampus CA2. Social rank affected cACC correlations with MDT, lateral habenula (LH), nucleus of the diagonal band (NDB), lateral septum (LS), and nucleus accumbens (ACB) (SOM Fig. 2B). These activity-based correlations are supported by previous neuroanatomical tracing studies: MDT directly connects with ACC, PL, and OFC ^31^, and ACC directly connects with NDB, LS, and MDT ^32,33^.

The MDT is primarily composed of excitatory projection neurons and lacks local inhibitory interneurons^34,35^. Accordingly, we found that *Fos*-positive cells in rank-1 and rank-4 were uniformly *Slc17a6-*positive (i.e., VGLUT2-positive) glutamatergic neurons (MDT^Glut^, Fig. 2B, C & SOM Fig. 2C). Together, these results indicate that social rank is associated with distinct patterns of neural activity, particularly in MDT^Glut^ and cACC neurons.

### MDT facilitates the establishment of hierarchy

Stable hierarchy emerges after multiple territorial interactions (Fig. 1C–F), suggesting underlying experience-dependent processes. To further address the role of MDT in social hierarchy formation and maintenance, we lesioned this structure either before or after hierarchy formation using targeted excitotoxic NMDA (N-methyl-D-aspartate) injections, which eliminates cell bodies but not fibers of passage^36^ (Fig. 2D, E).

In “naïve” cohorts, all four mice per group received lesions prior to hierarchy formation, whereas in “experienced” cohorts all four mice received lesions after hierarchy formation. Animals receiving sham surgeries with vehicle injections served as controls for both cohorts (Fig. 2D). Lesions did not affect aggressive behavior during resident-intruder assays tested in naïve cohorts (SOM Fig. 2D). However, compared to experienced and sham cohorts, naïve groups with MDT lesions displayed more variable Elo ratings, reduced hierarchy stability and pairwise consistency (Fig. 2F–I and SOM Fig. 2E–F). Lesions also resulted in longer post-operative tube test decision timing in naïve and experienced groups (SOM Fig. 2G–H).

We next tested whether MDT was required for the odor response to rank-1 urinary cues observed in swabbing experiments. In sham surgery control groups, rank-4 males swabbed with rank-1 urine had increased performance (SOM Fig. 2I) as previously demonstrated. By contrast, in MDT lesion groups, rank-4 performance was unaffected by urine swab (SOM Fig. 2K–L), suggesting that MDT is required for appropriate hierarchy-dependent responses to urinary olfactory cues.

Next, we investigated whether MDT more widely contributes to sociability or social recognition using a three-chamber assay and found no differences in the behavior of sham vs lesion (both naïve and experienced) animals (SOM Fig. 2M–N). Together these results suggest that, although MDT is not required for sociability nor social recognition, it is required for establishing a stable hierarchy and consistent interactions.

### MDT activation increases competitive performance

**Figure 3.**
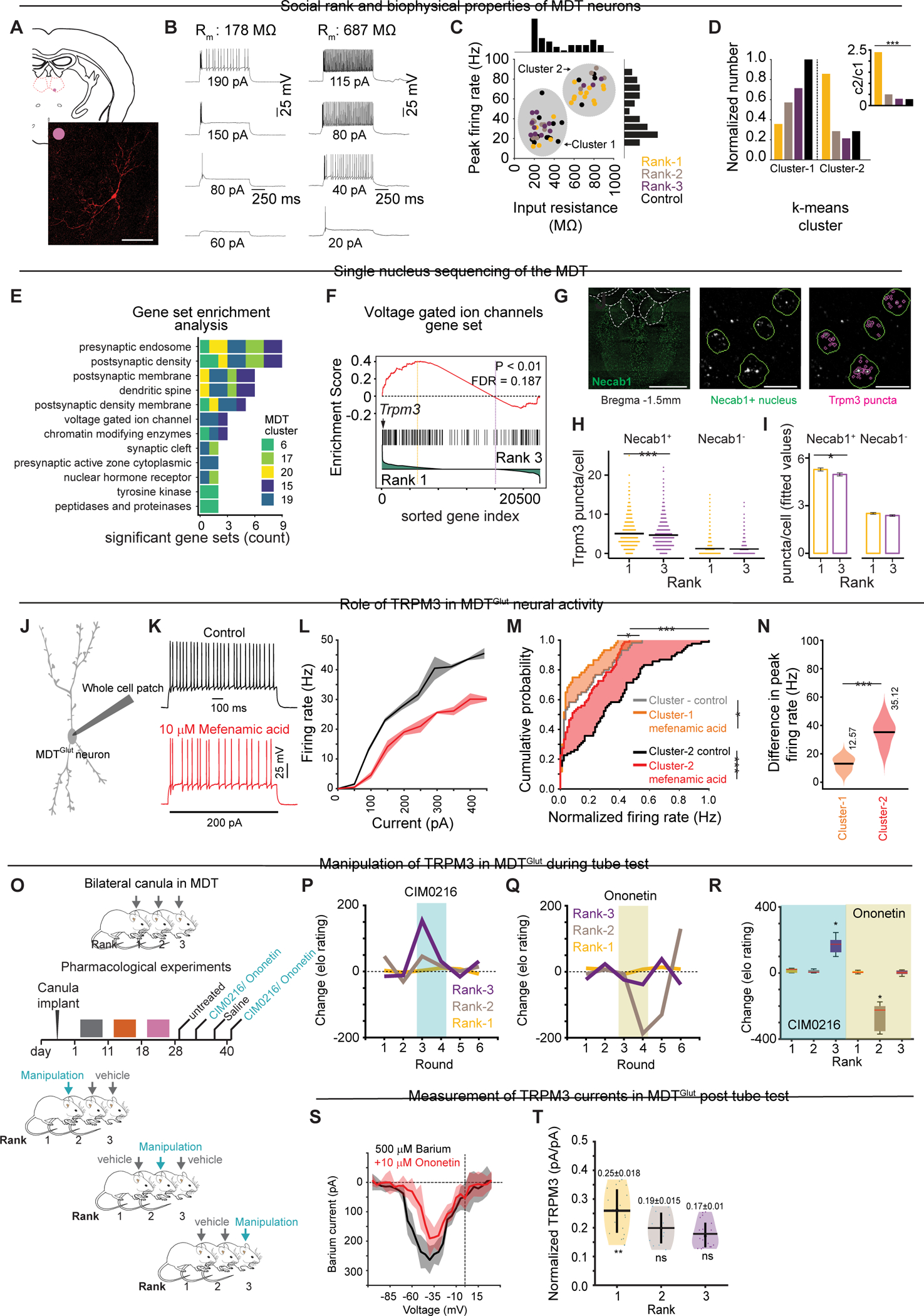
*Trpm3* expression in MDT is associated with social rank. **(A)** Example recorded MDT cell. **(B)** Example traces of cluster-1 (left) and cluster-2 (right) neurons. R_m_, input resistance. **(C)** Neuronal peak firing rate *vs*. input resistance by cluster-type (N=33 cluster-1 neurons; N=27 cluster-2 neurons). Histograms show bimodality for resistance and firing rate. **(D)** Effect of social rank on cluster-type proportions. Inset: ratio of cluster-2/cluster-1 neurons by rank (p<0.001). **(E)** Gene sets affected by social status by MDT cluster identity. X-axis: each count is one gene set per cluster per comparison (rank-1 vs. control; rank-3 vs. control, rank-1 vs. rank-3). **(F)** GSEA: Voltage gated ion channels (black vertical lines) sorted by differential expression between rank-1 (left) and rank-3 (right). Enrichment score (red line) is cumulative sum (left to right) that increases as VGIC genes are encountered and decreases when they are not. Genes left of yellow line (peak enrichment score) are upregulated in rank-1; genes right of purple line downregulated in rank-3. Correlation of gene expression with social rank (green; bottom). Permutation test corrected for multiple testing (FDR), p < 0.01. **(G)** Left and middle: Spatial distribution of) *Necab1*+ nuclei (green outlines) and *Trpm3* (white speckles and magenta outlines). Right: *Trpm3* puncta counts in *Necab1*+ (N = 35,865) and *Necab1*− (N = 69,530) cells. *Trpm3* counts shown as raw data **(H)** or fitted values from LMER **(I)**. **(J-N)** Current-clamp recordings in the MDT. **(K)** Example firing patterns in control or TRPM3 antagonist (mefenamic acid) solutions. **(L)** MDT frequency/current curves and **(M)** cumulative probability distribution of firing rates under control/antagonist conditions for cluster-1 (p=0.03) and cluster-2 (p<0.001) neurons. **(N)** Decrease in peak firing rates in cluster-1 and cluster-2 (N = 5 for cluster-1 and N = 6 for cluster-2, p<0.001). **(O-R)** Pharmacological manipulation of TRPM3 by bilateral cannulas. **(O)** Experimental strategy. **(P-Q)** TRPM3 activation (CIM0216) and inactivation (ononetin) *vs*. competitive performance during tube test. **(R)** Summary of activation and inactivation on competitive performance. N = 2 groups. **(S)** Mean voltage-current values of MDT neurons with sodium/potassium blockers (black), plus TRPM3 antagonist ononetin (red). **(T)** Rank-dependent fractional contribution of TRPM3 mediated barium current. (n=19, 12, 17 neurons for rank-1, rank-2, and rank-3, respectively). * p<0.05. ** p<0.01. ***p<0.001. Hartigans’ dip test: panel C. Wilcoxon rank-sum test: panels D, N, R and T. Kolmogorov Smirnov test: panel M. Data are mean ± s.e.m..

MDT *Fos* expression was associated with winning in tube test, prompting us to examine how chemogenetic manipulation of MDT^Glut^ neurons affects competitive performance. Separate cohorts of four *Vglut2*-Cre mice were bilaterally injected in MDT with conditional AAVs encoding either inhibitory or excitatory DREADDs (i.e., AAV-hSyn-DIO-hM3D(Gq)-mCherry or AAV-hSyn-DIO-hM4D(Gi)-mCherry) (Fig. 2J) and allowed to establish hierarchies. DREADD ligand CNO^37^ was delivered according to a repeated on-off schedule (Fig. 2K). In initial assays, we tested CNO injections in rank-1 and rank-4 mice in the absence of DREADDs and found that CNO alone did not affect the established hierarchy in control groups of C57BL/6J mice (SOM Fig 3A).

In cohorts undergoing chemogenetic excitation of MDT^Glut^ neurons, CNO transiently caused a robust competitive performance spike in rank-4 males (Fig. 2L–N) (SOM Video S1 of tube test) but did not affect rank-1 males (Fig. 2N). By contrast, MDT^Glut^ inhibition did not affect competitive performance of either high- or low-ranked males (SOM Fig 3B–D). In light of the lesion experiments described earlier, these results suggest MDT plays a role in hierarchy establishment and provides a signal that increases competitive performance of low-rank males within established hierarchies.

### Biophysical and molecular features of MDT neurons

To assess if social rank affects intrinsic MDT neuronal properties, we performed whole-cell patch clamp recordings in acute slices from individuals in established hierarchies (groups of three) and isolated controls. We recorded from MDT cell bodies in current-clamp mode and measured evoked spiking activity by injection of variable amounts of current (Fig. 3A). MDT neurons showed a bimodal distribution of peak firing rates and input resistances (Fig. 3B), confirmed by Hartigans’ dip test (p = 0 for both parameters). K-means clustering revealed two distinct populations: Cluster-1 (62%, 37/60 neurons) characterized by low firing frequency (<65 Hz) and low input resistance (<500 MΩ), and cluster-2 (38%, 23/60 neurons) characterized by high firing frequency (>50 Hz) and high input resistance (>500 MΩ) (Fig. 3C). Cluster-2 cells were predominantly found in rank-1 males, while cluster-1 cells were prevalent in rank-2, rank-3, and control males (Fig. 3C–D). Rank-1 animals showed a five-fold higher cluster-2 ratio compared to other ranks, indicating MDT neurons exhibit distinct excitability states corresponding to social rank.

We next sought to identify mechanisms underlying rank-dependent differences in MDT neuronal membrane properties by assessing the cellular and molecular composition of midline thalamic nuclei. We performed single nucleus RNA-seq (sNuc-Seq) on 33,582 neurons from rank-1, rank-3, and control mice (10x Chromium platform)^38,39^. Five distinct MDT cell populations clustered according to transcriptional similarities. These MDT clusters expressed the excitatory marker *Slc17a6* (VGLUT2), accounted for 12,782 nuclei (average of 4,699 transcripts and 2,169 unique genes per nucleus), and were separable into two groups according to transcriptional relationships and anatomy inferred from expression of key marker genes (SOM Fig. 3E–G). In the first group, two clusters associated with *Necab1* expression were positioned in a ring encompassing the MDT, PVT, and intralaminar nuclei (“ring cells”) (SOM Fig. 3G,H), consistent with two recent studies ^40,41^. In the second group, three clusters associated with *Kcnip3* expression were positioned within the central compartment of the MDT (“body cells”) (SOM Fig. 3G).

We next asked how hierarchy formation affected transcription in MDT clusters using gene set enrichment analysis (GSEA)^42,43^. We tested for differential expression of gene sets or biological pathways (N = 145 gene sets) within MDT clusters (N = 5) for each of three pairwise comparisons (rank-1 vs. control; rank-3 vs. control; and rank-1 vs. rank-3), yielding a total of 2,175 tests. Biological pathways that were significantly affected in two or more tests (N=61/2,175) were found in MDT “ring” and “body” clusters and enriched for genes associated with pre- and post-synaptic structures (46/61, 75%), cellular enzymes including chromatin modifiers and CMGC kinases (16%), voltage gated ion channels (5%), and nuclear hormone receptors (2%) (Fig. 3E).

### *Trpm3* expression in MDT correlates with social rank

To further identify rank-dependent gene expression differences, we analyzed the intersection of GSEA results with genome-wide differentially expressed genes (DEGs) for each pairwise rank comparison on a per-cluster basis, which uncovered genes that were significant for both GSEA and DEG tests. The strongest effects of rank-dependent gene expression were found in the *Necab1-*positive ring clusters. Over 80% of *Fos*+ cells following tube tests were *Necab1*+ (SOM Fig. 3I), indicating this population’s specific recruitment during social competition. Differentially expressed genes in *Necab1*+ clusters between ranks were enriched for voltage gated ion channels (Fig. 3F) and tyrosine kinases (SOM Fig. 3J–K). Five ion channel genes showed higher expression in rank-1 animals (*Mcoln2*, *Trpm3*, *Kcnh8*, *Scn3a*, *Cacna1b,* SOM Fig. 3J).

Prompted by their known contribution to membrane excitability, we next sought to validate differential expression of *Trpm3* and *Scn3a* voltage gated ion channel genes using quantitative RNA FISH and confirmed an effect of rank on *Trpm3* expression. TRPM3 is a transient receptor potential (TRP) ion channel activated by changes in membrane voltage, temperature, and endogenous neurosteroids ^44^. mRNA expression of *Trpm3* in *Necab1* cells was readily detectable as single molecule FISH puncta (SOM Fig. 3L) and, as predicted from sNuc-Seq data, *Trpm3* puncta counts in *Necab1*+ cells were higher in rank-1 compared to rank-3 mice (Fig. 3 G–I). There was no such difference in *Necab1*-negative cells (Fig. 3H–I). Thus, regulation of *Trpm3* channel expression in the MDT is rank-dependent.

### TRPM3 shapes neural excitability and social rank

We next investigated how TRPM3 may influence the firing rate of MDT^Glut^ neurons using current-clamp recordings and pharmacology in untrained naïve animals, directing our recordings towards MDT ring cells. TRPM3 is a non-selective cation channel permeable to Ca^2+^ and, to a lesser extent, K^+^, and Na^+ 44,45^. We first examined cell-intrinsic properties. Blocking TRPM3 function with the antagonist mefenamic acid ^46^ resulted in decreased firing rates of cluster-1 and cluster-2 neurons (Fig. 3J– N). Because mefenamic acid inhibits COX-2 ^47^, which could generate confounding effects, subsequent experiments used ononetin, a more selective TRPM3 antagonist ^48^. Compared to control solution, bath application of 10 μM ononetin, as well as mefenamic acid, reduced MDT neuron firring rate (SOM Fig. 3M–N). The two antagonists had similar effects on the FI curves (firing rate of neurons plotted as a function of input current) and peak firing rates of MDT neurons (SOM Fig. 3N).

We next addressed whether pharmacological manipulation of TRPM3 *in vivo* affected competitive performance. Mice in established hierarchies received bilateral, cannulated MDT injections (500 nL) of TRPM3 agonist (CIM0216) or antagonist (ononetin) according to an on-off schedule (Fig. 3O). The agonist significantly improved rank-3 competitive performance without affecting rank-1 or rank-2 animals. Conversely, the antagonist reduced rank-2 competitive performance but had no effect on rank-1 and rank-3 animals (Fig. 3P–R and Video S2). Thus, *in vivo* manipulation of the TRPM3 channel in MDT modulates competitive performance.

We next examined how TRPM3 contributes to rank-dependent firing rates of MDT^Glut^ neurons through *ex vivo* patch clamp recordings. We quantified TRPM3 calcium currents using barium (500 nM) as a charge carrier with depolarizing voltage-clamp protocols. The antagonist ononetin reduced barium currents in control slices (Fig. 3S and SOM Fig.3 O–P) ^49,50^. We then evaluated barium currents with and without ononetin by rank. Barium currents were larger in rank-1 compared to rank-2 and 3 males (SOM Fig. 3P). The peak amount of TRPM3-mediated barium current (normalized by peak current) was reduced by 25%, 19% and 17% in rank-1, rank-2 and rank-3, respectively (Fig. 3T), with current spread also reduced (standard deviation changing from 0.0387 pA/pA in controls to 0.0764 pA/pA in rank-1). These results indicate that calcium currents substantially contribute to TRPM3-mediated neuronal firing in MDT^Glut^ neurons, particularly in high-rank males, identifying how TRPM3 activation may increase competitive performance while inhibition decreases it.

### MDT inputs: organization and synaptic properties

**Figure 4.**
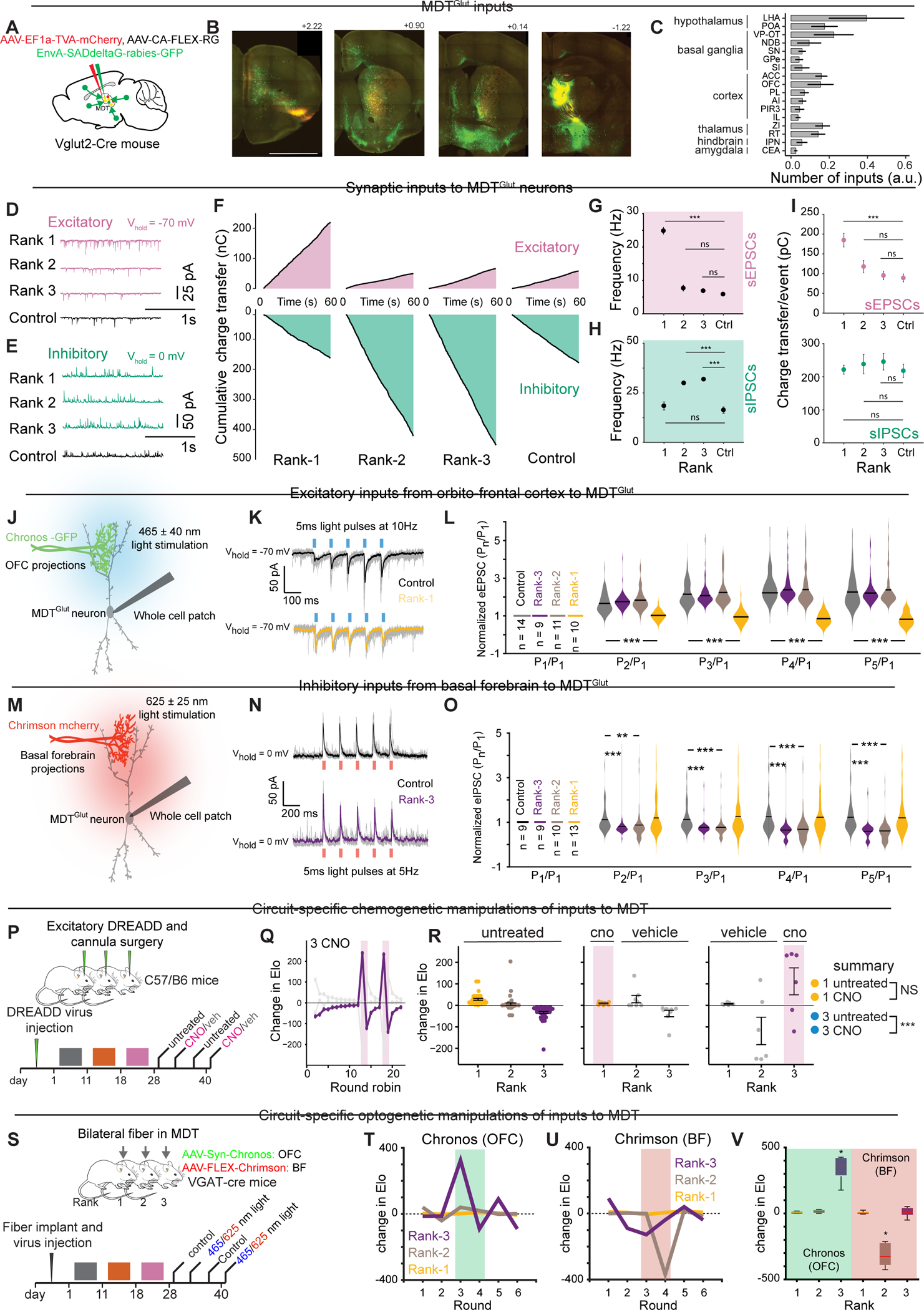
MDT Inputs: organization and synaptic properties. **(A)** Monosynaptic retrograde tracing from MDT^Glut^ neurons. **(B)** Input areas displaying rabies-positive neurons. **(C)** Quantification of inputs to MDT^Glut^ neurons (convergence index multiplied by the percentage of MDT starter cells), N = 3 mice. **(D-I)** Whole-cell voltage-clamp recordings of synaptic activity in MDT^Glut^. Example sEPSCs **(D)** and sIPSCs (**E**). Example per-minute cumulative charge transfer of sEPSCs and sIPSCs **(F)**. Frequency of sEPSCs (N = 53 neurons) **(G)**. Frequency of sIPSCs (N = 43 neurons) **(H)**. Charge transfer of sEPSCs/sIPSCs, (N = 53 excitatory event; N = 43 inhibitory event) **(I)**. **(J)** Electrophysiological recordings during optogenetic activation of OFC→MDT projections. **(K)** Top: eEPSCs from control mice (grey: 10 trials, black: mean response of 20 consecutive trials) during OFC→MDT activation (light pulses: 5 ms duration, 465 nm at 10 Hz). Bottom: same paradigm in rank-1 animals; mean response (yellow). **(L)** Summary showing control, rank-2, and rank-3 display paired pulse facilitation while rank-1 displays paired pulse depression (N = 9 animals). **(M)** Electrophysiological recordings during optogenetic activation of BF→MDT projections. **(N)** Top: eIPSCs from control mice (grey: 5 trials, black: mean response of 10 consecutive trials) during BF→MDT activation (light pulses: 5 ms duration, 625 nm at 5 Hz). Bottom: same paradigm in rank-3 animals; mean response (purple). **(O)** Summary for control, rank-1, rank-2, and rank-3 animals (N = 8 animals). **(P)** DREADD manipulation of OFC→MDT projections and timeline. **(Q)** Example competitive performance during CNO delivery to rank-3 mouse. **(R)** Competitive performance *vs*. final tube rank in untreated mice (left). Effect of CNO delivery to rank-1 (middle) or rank-3 (right). **(S)** Optogenetic manipulation of OFC and BF projections to MDT and timeline. **(T)** Effect of activation of OFC→MDT and **(U)** BF→MDT projections on competitive performance by rank. **(V)** Summary of OFC→MDT and BF→MDT activation on competitive performance. N = 2 groups. * p<0.05. ** p<0.01. ***p<0.001. Wilcoxon rank-sum test: panels G, H, I, L, O and V. Data are mean ± s.e.m.).

Having characterized rank-dependent, cell-intrinsic properties of MDT^Glut^ neurons, we next examined non-cell autonomous synaptic changes. We visualized brain-wide inputs to MDT^Glut^ neurons in *Vglut2*-ires-Cre mice using (G)-deleted rabies-based retrograde trans-synaptic labeling^51^ (Fig. 4A and SOM Fig. 4A–B). MDT^Glut^ neurons receive mono-synaptic inputs from nearly 20 brain regions, including hypothalamic areas (e.g., lateral hypothalamus (LHA), POA), basal forebrain areas (e.g., NDB, ventral pallidum (VP), substantia innominata (SI)), deep-layer cortical areas (ACC, OFC, PL), olfactory areas (piriform cortex, olfactory tubercle, OFC), and intra-thalamic areas (reticular thalamus and zona incerta) (Fig. 4B–C).

Next, we investigated rank-dependent spontaneous synaptic inputs to MDT neurons using voltage-clamp recordings, with the majority (53/73) of recordings in the MDT ring (SOM Fig. 4C). Spontaneous excitatory and inhibitory post-synaptic currents (sEPSCs and sIPSCs) were measured at −70 mV and 0 mV (Fig. 4D–E). MDT neurons of rank-1 males received more excitation than rank-2, rank-3, and control males, and received less inhibition than rank-2 and rank-3 males (Fig. 4F). Relative to individually housed controls, rank-1 MDT neurons received more frequent and larger sEPSCs, whereas rank-2 and rank-3 males were similar to controls (Fig. 4G, I). By contrast, sIPSC frequency was higher in rank-2 and rank-3 males compared to controls, while rank-1 males were similar to controls (Fig. 4 H). There was no effect of social rank on the sIPSC size (Fig. 4I). Thus, strength and frequency of synaptic inputs to MDT neurons is rank-dependent.

To identify rank-dependent inputs to MDT, we next assessed synaptic plasticity of functional connectivity using optogenetics and whole-cell voltage-clamp recordings. Short-term plasticity reflects changes in synaptic strength caused by patterns of presynaptic firing activity and is critical for information processing^52^. Short-term plasticity can be measured through paired-pulse responses and is observed as increased or decreased strength in the first pulse relative to later pulses (i.e., facilitation or depression). CTB-retrograde labeling combined with FOS immunohistochemistry identified four potential presynaptic regions active during tube tests: piriform cortex, lateral preoptic area, OFC, and basal forebrain (data not shown). We injected channelrhodopsin AAVs expressing Chronos (465nm light stimulation) or Chrimson (625nm light stimulation) into these presynaptic areas and recorded optically evoked postsynaptic eEPSCs (−70 mV) and eIPSCs (0 mv) in MDT neurons using five 5-ms pulses (5 or 10 Hz) (SOM Fig. 4D–E). Only OFC→MDT and BF→MDT projections produced significant evoked responses (Fig. 4J–K, M–N), with no substantial activity from piriform cortex or lateral preoptic projections.

We found rank-dependent release probability^53^ for OFC and BF inputs (Fig. 4J–O). OFC projections to MDT (i.e., OFC→MDT; SOM Fig. 4F) exhibited eEPSCs (but not eIPSCs) and showed significant facilitation (i.e., lower probability of release) across stimulation pulses in control and lower rank (rank-2 and rank-3) compared to rank-1 animals (Fig. 4J–K). By contrast, OFC→MDT synapses in rank-1 animals showed significant depression (i.e., higher probability of release) across stimulation pulses (Fig. 4K–L). These data suggest a higher probability or “potentiation” of release from OFC→MDT axon terminals in rank-1 animals.

Basal forebrain (BF, including the NDB and substantia innominata) inputs to MDT (i.e., BF→MDT; SOM Fig. 4F) included eEPSCs and eIPSCs with distinct synaptic properties. Inhibitory BF→MDT inputs showed depression across pulses in lower ranks (ranks 2 and 3) compared to control and rank-1 animals (Fig. 4M–O), suggesting that inhibitory BF→MDT inputs are potentiated in lower ranks. By contrast, BF→MDT excitatory projections also showed paired pulse depression, but independent of social rank (SOM Fig. 4G–I). Thus, we observed concurrent potentiation of excitatory OFC→MDT inputs in high-ranks and of inhibitory BF→MDT inputs in low ranks.

### *In vivo* manipulation of OFC→MDT and BF→MDT inputs.

We next assessed whether experimental manipulations of OFC→MDT and BF→MDT projections affect competitive performance *in vivo*. Separate cohorts of three C57BL/6J mice were injected with excitatory or inhibitory DREADD AAVs (i.e., AAV-hSyn-hM3D(Gq)-mCherry or AAV-hSyn-hM4D(Gi)-mCherry) into OFC and bilateral cannulas were implanted in MDT for CNO infusion (Fig. 4P, SOM Fig. 4J). Repeated chemogenetic manipulations were made according to an on-off schedule: during “on” days, CNO was infused to MDT of the tested animal while the other animals received vehicle. Chemogenetic activation of OFC→MDT caused a robust spike in the competitive performance of rank-3 mice but did not affect the performance of rank-1 animals (Fig. 4Q,R). By contrast, chemogenetic inhibition of OFC→MDT did not affect competitive performance (data not shown; SOM Fig. 4K).

In a second set of experiments, we used optogenetics to manipulate both OFC→MDT excitatory projections and BF→MDT inhibitory projections in the same animal, which were potentiated in high-rank and low-rank animals, respectively (Fig. 4S). To target both pathways simultaneously *in vivo*, *Vgat*-Cre mice received bilateral injections of conditional Chrimson (AAV-hSyn-FLEX-ChrimsonR-tdTomato) in BF and non-conditional Chronos (AAV-hSyn-Chronos-GFP) in OFC, with optic fibers in MDT (Fig. 4S). Activating OFC→MDT significantly increased rank-3 competitive performance without affecting rank-1 or rank-2 animals (Fig. 4T,V), consistent with chemogenetic manipulations. By contrast, activating *Vgat-Cre*-positive BF→MDT projections decreased rank-2 competitive performance (Fig. 4U–V and Video S3) without affecting rank-1 or rank-3. Together, these results suggest that excitatory OFC→MDT and inhibitory BF→MDT synaptic connections undergo plasticity to regulate competitive performance within hierarchies.

### MDT outputs and function of MDT→cACC projections

**Figure 5.**
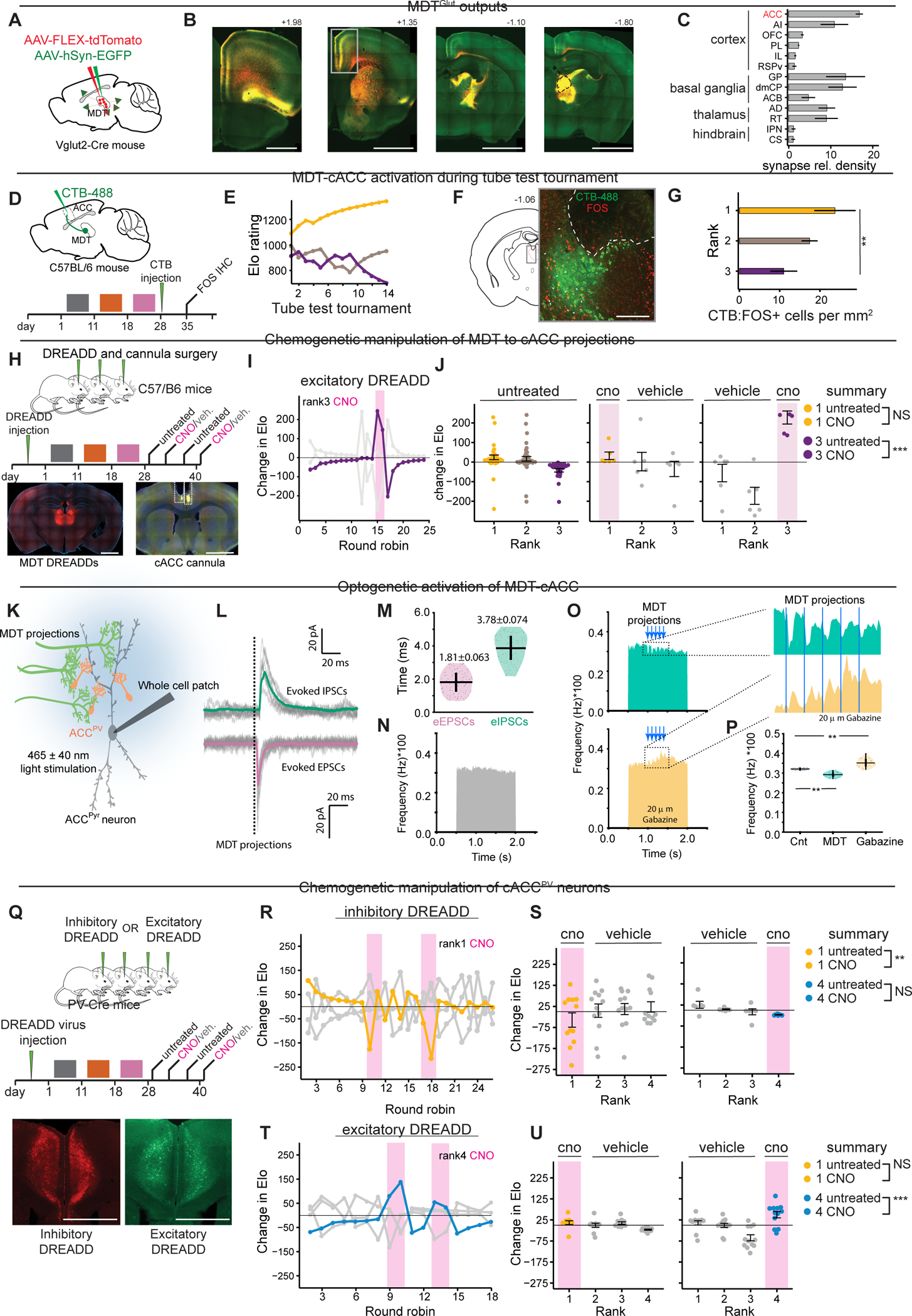
cACC excitation-inhibition balance is influenced by MDT inputs and regulates competitive performance. **(A-C)** Anterograde tracing of MDT^Glut^ neurons. Tracing scheme **(A)**; afferent projection synapses (green) and fibers (red) in control male **(B)**; relative density of synapses **(C)**; N = 3 mice. **(D)** MDT→cACC tracing scheme. **(E)** Example Elo ratings from trio prior to FOS labeling. **(F)** Labeling of CTB, FOS IHC, and co-labeling (white arrows) in rank-1 mouse. **(G)** CTB-FOS+ cell counts *vs*. rank (N = 12 mice). **(H)** DREADD expression in MDT neurons and cannulas in cACC and timeline. **(I)** Competitive performance of rank-3 male receiving CNO. **(J)** Competitive performance *vs*. final tube rank in untreated mice (left). Effect of CNO on rank-1 (middle) or rank-3 (right) mice. **(K-P)** Optogenetic activation of MDT→cACC projections and cACC^Pyr^ firing rate. **(K)** cACC^Pyr^ voltage-clamp recordings and activation of MDT projections. **(L)** Example evoked IPSCs (top) / EPSCs (bottom) in cACC^Pyr^ following MDT→cACC activation. **(M)** eIPSC and eEPSC latencies (N=9 neurons, p<0.01). **(N)** Stable firing rate in cACC^Pyr^ by injection of noise patterns. **(O)** MDT→cACC activation results in brief dip in stable firing rate of cACC^Pyr^ (top) that is blocked by GABA receptor antagonist Gabazine (bottom). **(P)** Summary of effects on excitation-inhibition balance in cACC^Pyr^ neurons (N=11 neurons). **(Q)** DREADD expression in cACC of PV-Cre mice and timeline (top and middle). Histology of inhibitory/excitatory DREADDs in cACC (bottom). **(R)** Competitive performance of rank-1 male receiving CNO. **(S)** Competitive performance *vs*. CNO delivery to rank-1 (left) or rank-3 (right) mice, N = 3 groups. **(T-U)** Effect of excitation of cACC^PV^ on social rank, N = 3 groups. LMER: panels G, S and T. * p<0.05. ** p<0.01. ***p<0.001. Wilcoxon rank-sum test: panels M and P. Data are mean ± s.e.m..

Next, we sought to define and characterize the outputs of MDT^Glut^ neurons. Projections from MDT^Glut^ neurons were visualized using two combined AAVs to drive expression of reporter fluorophores in both axons (tdTomato) and presynaptic terminals (Synaptophysin-GFP) (Fig. 5A–B). As previously described, the majority of MDT^Glut^ outputs were to layer II/III areas of the dmPFC (including the infralimbic cortex (IL), PL, and rostral ACC) and other cortical areas like OFC, retrosplenial cortex, and agranular insular area ^54,55^. Additional targets included basal ganglia areas (i.e., globus pallidus, dorsomedial caudate putamen, and nucleus accumbens)^56^. The ACC, from its most rostral (> bregma +2.0) to caudal (< bregma − 2.0) coordinates, received the greatest fraction of inputs (Fig. 5C).

We next investigated the cACC as a major target of MDT^Glut^ projections. Rank-1 males showed decreased *Fos* expression in cACC but increased expression in MDT after tube tests (Fig. 2A). Since MDT projections can drive feedforward inhibition of frontal cortical areas via parvalbumin-expressing (PV) interneurons ^54,57–59^, we hypothesized that MDT may inhibit cACC during competition. Retrograde tracing from cACC PV cells (cACC^PV^) confirmed this connectivity (SOM Fig. 5A–C). While the majority of presynaptic inputs to cACC^PV^ were local (~79% of inputs), we also detected input cells in the lateral MDT ring (~5%, SOM Fig. 5B–C), the same MDT subdivision showing rank-dependent *Fos* expression (Fig. 2C) and transcriptional changes (Fig. 3E–I). Confocal imaging revealed close apposition of GFP-labelled MDT axon swellings (AAV-hSyn-Chronos-GFP) to tdTomato-labelled cACC^PV^ cell bodies in a reporter line (SOM Fig. 5D).

We next addressed whether MDT projections to cACC (MDT→cACC) functionally relate to social rank. After injecting retrograde tracer CTB into cACC of mice in established hierarchies, we measured FOS immunostaining in MDT following tube tests (Fig. 5D,E). Rank-1 mice had more double-labeled CTB-FOS cells than rank-3 mice in the lateral MDT (Fig. 5F,G). Next, we tested the functional role of MDT→cACC projections by injecting non-conditional AAVs expressing excitatory and inhibitory DREADDs (i.e., AAV-hSyn-hM3D(Gq)-mCherry or AAV-hSyn-hM4D(Gi)-mCherry) into MDT and implanting bilateral cannulas over the cACC for CNO infusion (Fig. 5H). Activating MDT→cACC projections increased the competitive performance of rank-3 animals but had no effect on rank-1 (Fig. 5I–J). There was no effect of chemogenetic inhibition (data not shown).

The use of excitatory DREADDs to activate MDT→cACC projections may result in back-propagation of action potentials that could, in turn, activate other brain regions such as the dmPFC. To address this issue, we used a multi-virus chemogenetic strategy to activate MDT→cACC projections while simultaneously inhibiting pyramidal cells of the dmPFC (SOM Fig. 5D–E). We found that rank-3 males receiving MDT→cACC excitation with concurrent dmPFC inhibition displayed improved competitive performance (SOM Fig 5F). These results recapitulate chemogenetic manipulations of MDT^Glut^ soma (Fig. 2K–N), and cannulated activation of MDT→cACC projections (Fig. 5 H–J), suggesting that MDT→cACC activity is sufficient to increase competitive performance in lower ranking individuals.

Next, we performed optogenetic assisted circuit mapping to directly test the hypothesis that MDT may drive inhibition of cACC. We expressed Chronos (AAV-hSyn-Chronos-GFP) in MDT neurons and performed *ex vivo* whole cell voltage clamp recordings in cACC (Fig. 5K). We recorded eIPSCs and eEPSCs in cACC pyramidal cells (i.e., cACC^Pyr^ ) during light-stimulation of MDT→cACC projections in control animals. We detected eIPSCs in all recorded cACC^Pyr^ cells, and eEPSCs in a subpopulation (71%) (Fig. 5L). eIPSC latency was significantly longer than eEPSCs latency (3.78ms vs. 1.81ms, Fig. 5M), suggesting that MDT→cACC projections display direct excitatory, and indirect inhibitory, synaptic connections to cACC^Pyr^.

Next, we evaluated how MDT→cACC firing affects excitation-inhibition balance in cACC. We used a “white noise” paradigm to generate stable neuronal firing patterns in cACC^Pyr^ neurons and to quantify the precise contribution of specific inputs to the responses of those neurons ^60^ (Fig. 5N and SOM Fig. 5H,I). Light-stimulation of MDT→cACC resulted in brief but significant reductions in stimulus-induced cACC^Pyr^ firing rates (Fig. 5O). Blocking GABAergic modulation (20 μM gabazine) reversed this effect and increased firing rate upon stimulation (Fig. 5 O–P). These results demonstrate that despite evoking excitatory and inhibitory events, MDT→cACC inputs produce net inhibition of cACC^Pyr^ activity via GABA_A_ receptors, supporting the notion that *Trpm3*-mediated high firing of MDT^Glut^ neurons in high-rank animals reduces cACC^Pyr^ activity.

### cACC^PV^ neurons regulate competitive performance

Our observations that MDT→cACC activation results in cACC^Pyr^ inhibition and modulation of competitive performance, and that cACC *Fos* is anti-correlated with rank prompted us to test whether cACC^PV^ neuronal activity alone may modulate rank. We predicted that inhibition (or excitation) of cACC^PV^ neurons would decrease (or increase) competitive performance. We injected conditional DREADD AAVs (AAV-hSyn-DIO-hM3D(Gq)-mCherry and AAV-hSyn-DIO-hM4D(Gi)-mCherry) into the cACC in groups of four PV-Cre mice (Fig. 5Q). CNO-mediated DREADD inhibition of cACC^PV^ caused a pronounced, transient decline in competitive performance of rank-1 males but did not affect rank-4 males (Fig. 5R,S). By contrast, CNO-mediated DREADD excitation of cACC^PV^ caused a pronounced, transient competitive performance spike in rank-4 males, but did not affect rank-1 (Fig. 5T,U). Thus, cACC^PV^ excitation (or inhibition) increases (or decreases) competitive performance, demonstrating bidirectional control. These results are consistent with a model where MDT^Glut^ activation of cACC^PV^ cells increases rank, while inhibition of cACC^PV^ decreases rank.

### Excitation/inhibition of cACC/dmPFC in tube test

**Figure 6.**
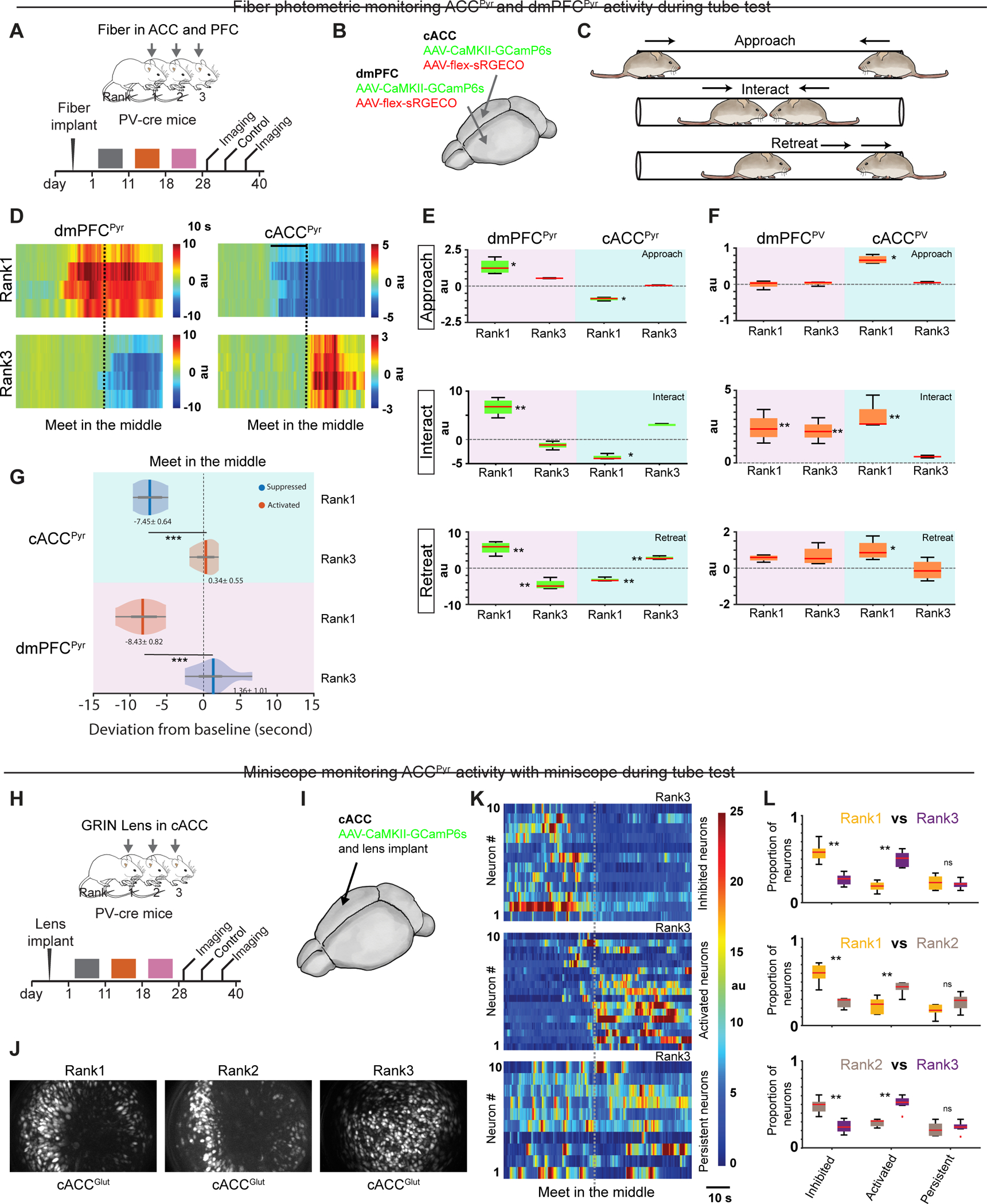
Rank-dependent calcium activity of prefrontal and cingulate cortical neurons during tube test. **(A)** Expression of GCaMP6s and sRGECO in cortex of PV-Cre mice and timeline. **(B)** Calcium indicators and fiber-photometry implants to record from PV and Pyr neurons in left dmPFC and right cACC. **(C)** Three phases of the tube test. **(D)** Pyramidal calcium activity in dmPFC / cACC during tube test**. (E-F)** Activity of pyramidal **(E)** and PV **(F)** neurons based on phase of the tube test. N=2 groups; asterisks signify differences from zero. **(G)** Timing of deviation in cACC^Pyr^ and dmPFC^Pyr^ baseline activity relative to meeting in the middle of tube (time 0) for rank-1 vs rank-3. **(H-K)** Activity of cACC^Pyr^ neurons during tube test. **(H)** GRIN lens implants and timeline. **(I)** Viral GCaMP6s expression in cACC^Pyr^ neurons in right cACC. **(J)** Maximum intensity projections of cACC^Pyr^ neuronal activity *vs*. rank during tube test. **(K)** Inhibited, activated, and persistent clusters of cACC neurons (10 representative neurons each) of rank-3 animals. **(L)** Fraction of neurons for each cluster type *vs*. competitive performance (calculated across all neurons). Number of neuronal ROIs: rank-1, 1729; rank-2, 1523; rank-3, 1568. * p<0.05. ** p<0.01. Wilcoxon rank-sum test: panels E, F, and L. Data are mean ± s.e.m..

Our data uncovered a pattern of excitation and inhibition in cACC that is opposite to that described in prelimbic dmPFC in cohoused groups^21^. We therefore investigated *in vivo* neural activity in both regions during tube tests. Using fiber photometry and AAV-driven calcium indicators, we monitored cACC and dmPFC activity in both pyramidal neurons (GCaMP6s expressed via the CaMKII promoter^61^) and PV interneurons (Cre-dependent sRGECO) of PV-Cre mice. Each mouse received dual optic fiber implants above the right cACC and left dmPFC (Fig. 6A–B). Calcium activity was monitored during three sequential phases of the tube test: approach, interaction, and retreat (Fig. 6C). In dmPFC^Pyr^ neurons, activity was higher in rank-1 than rank-3 males across all phases, consistent with previous findings ^21^. Conversely, cACC^Pyr^ activity was lower in rank-1 than rank-3 males throughout all phases (Fig. 6 D–E). In rank-1 males, changes in both dmPFC^Pyr^ and cACC^Pyr^ activity deviated from baseline during approach and several seconds prior to meeting in the middle, while in rank-3 males changes deviated from baseline only upon meeting in the middle and retreating (Fig. 6D–E).

We next analyzed the activity of PV interneurons. cACC^PV^ neurons in rank-1 animals showed sustained activity above baseline during all three phases of the tube test, while cACC^PV^ neurons in rank-3 animals did not show change during any phase (Fig. 6F). dmPFC^PV^ neurons increased activity during interaction in both rank-1 and rank-3 animals, but not during approach and retreat (Fig. 6F). These results point to highly divergent activity patterns in dmPFC/cACC pyramidal and PV neurons during tube test interactions, with high rank animals characterized by decreased cACC^Pyr^ and increased cACC^PV^ activity, versus increased dmPFC^Pyr^ neuronal activity.

Analysis of dmPFC^Pyr^ / cACC^Pyr^ activity across pairwise contests showed that losing was associated with activity changes at physical contact, while winning correlated with changes several seconds before contact (Fig. 6G and SOM Fig. 6A). During rank-1 wins, deviations appeared approximately seven seconds before contact with rank-3, and six seconds before contact with rank-2 animals (Fig. 6G and SOM Fig. 6B). When rank-2 defeated rank-3, deviations occurred approximately three to five seconds before contact (SOM Fig. 6C). Thus, winning is associated with simultaneous changes in dmPFC^Pyr^ / cACC^Pyr^ activity that precede physical contact.

In the next set of experiments, we investigated the activity patterns of individual cACC^Pyr^ neurons. Here, we injected AAV-CaMKII-GCaMP6s to label excitatory neurons, implanted a GRIN lens in cACC, and used miniscopes (Bruker Inscopix) to monitor neuronal activity during the tube test (Fig. 6H–J). cACC^Pyr^ neurons could be subdivided into three distinct clusters according to activity: (a) inhibited, (b) activated, and (c) persistent activity (i.e., unaffected during tube test interactions) (Fig. 6K, SOM Fig. 6D).

We then investigated how cACC^Pyr^ activity relates with social rank *vs.* the state of winning or losing—an analysis enabled by rank-2 mice that sometimes win and sometimes lose. Winning animals showed a greater fraction of inhibited to activated neurons, while losing animals displayed more activated than inhibited neurons (Fig. 6L, SOM Fig. 6D). For example, in rank-1 vs. rank-3 contests, rank-1 neurons were predominantly inhibited (18.7% activated vs. 58.2% inhibited), while rank-3 neurons were predominantly activated (50.3% activated vs. 26.8% inhibited). Analysis of rank-2 animals revealed that cACC^Pyr^ activity correlates more strongly with winning/losing state than social rank (Fig. 6L). When rank-2 defeated rank-3, neurons were predominantly inhibited (29.2% activated vs. 48.7% inhibited), but when rank-2 lost to rank-1, neurons were mostly activated (42% activated vs. 26.7% inhibited) (SOM Fig. 6D–E). Moreover, rank-2 cACC^Pyr^ neurons displayed early deviations from baseline during winning and late deviations during losing (SOM Fig.6B–C). Thus, cACC^Pyr^ activity patterns are strongly associated with winning and losing.

**Figure 7.**
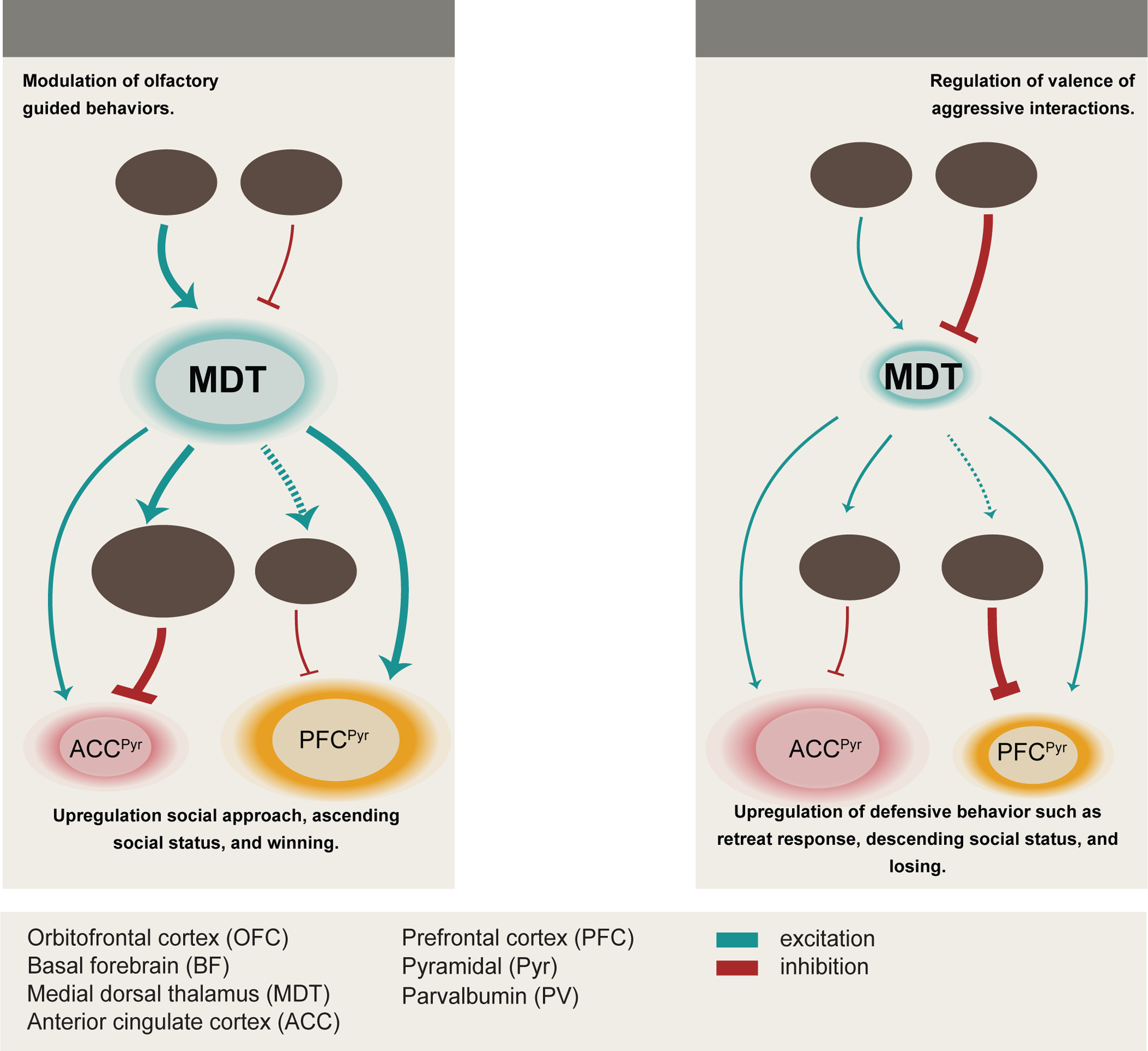
Model: Plasticity of Forebrain-MDT-cACC and Forebrain-MDT-mPFC circuits in social hierarchy behavior. MDT neurons in higher rank males show enhanced excitatory inputs from orbital frontal cortex (OFC) and reduced inhibitory inputs from basal forebrain (BF). MDT neurons send strong monosynaptic projections to parvalbumin-positive inhibitory neurons and weak monosynaptic projections to excitatory pyramidal cells of the cACC. Additionally, MDT neurons send strong projections to dmPFC pyramidal cells. Enhanced MDT activity in higher rank animals leads to feedforward inhibition of cACC and excitation of dmPFC pyramidal cells. By contrast, MDT neurons in lower ranks receive enhanced inhibition from BF and reduced excitation from OFC; reduced MDT activity leads to disinhibition of cACC pyramidal cells, and lower activity of dmPFC pyramidal cells.

These findings support a model where high-rank status is associated with enhanced MDT→cACC feedforward inhibition via cACC^PV^ neurons and low-rank status with disinhibition of the cACC^Pyr^ population. Moreover, winning is associated with simultaneous changes in dmPFC^Pyr^ and cACC^Pyr^ activity (Fig. 7).

## DISCUSSION

Our study demonstrates how competitive performance in territorially separated animals is regulated by plasticity in a multi-synaptic circuit. We identify MDT as a critical hub receiving OFC and BF inputs and projecting to cACC. Rank-dependent plasticity within this circuit is partly driven by cell-intrinsic changes in *Trpm3* expression in MDT^Glut^ neurons and by plasticity in BF→MDT and OFC→MDT synaptic inputs.

In high-rank animals, the MDT→cACC circuit is potentiated and linked to feed-forward inhibition of cACC^Pyr^, while in low-rank animals it is depressed and linked to cACC^Pyr^ excitation. Building on previous studies, we uncover coordinated but opposite patterns of MDT-mediated activity in dmPFC and cACC during competition: competitive performance can be increased by disinhibition of dmPFC^Pyr 21^ or by feedforward inhibition of cACC^Pyr^ (Fig. 7).

The MDT functions as a relay center and information transformer regulating cortical interactions ^35^. High-rank males show more excitable MDT neurons, potentiated OFC→MDT excitatory inputs, and weaker BF→MDT inhibitory inputs compared to low-rank males. We also show that high-rank males express more *Trpm3* in MDT ring cells; TRPM3 regulates MDT neuronal excitability through calcium currents; and TRPM3 manipulation regulates competitive performance. This suggests that TRPM3 strengthens MDT→cACC feedforward inhibition and competitive ability, potentially alongside other channels.

Previous work showed that hierarchies in cohoused males are regulated by MDT projections to dmPFC, where winning is triggered by dmPFC^Pyr^ excitation, dmPFC^PV^ inhibition, or MDT→dmPFC stimulation ^15,19–21^. While supporting this feedforward *excitatory* model, we reveal a parallel feedforward *inhibitory* model where increased MDT→cACC activity in high-rank males increases cACC^PV^ activity, decreases cACC^Pyr^ activity, and enhances competitive performance; a model supported by other studies showing MDT→ACC projections activate ACC^PV 54,57^ and inhibit ACC^Pyr 59^.

The ACC has diverse functions ^62^. Relevant to our study, ACC^PV^ activity encodes longer wait times during foraging ^63^ and reduces fearful behavior ^64^, while ACC^Pyr^ activity regulates threat avoidance^65 65,66^. We suggest that MDT instructs cACC to modulate responses to conspecifics during competition, and that the contrasting activity patterns in cACC and dmPFC may be essential for consolidating competitive outcomes. For example, in high-rank animals, elevated dmPFC activity might drive social approach ^67^ while lowered cACC activity might blunt threat perception ^65^. That dmPFC^Pyr^ and cACC^Pyr^ neurons display synchronicity during competition (Fig. 6G) suggests both modulate status.

Throughout our experiments, MDT activation increased performance of low-rank males, while inhibition had milder effects. Notably, cACC^PV^ inhibition was uniquely effective at reducing rank-1 performance (cACC^PV^ excitation also increased lower ranks’ performance ). A plausible interpretation is that, during hierarchy formation, MDT→cACC activity stabilizes excitatory-inhibitory balance in the cACC; once hierarchy is established, cACC^PV^ neurons become a control hub for both ascending and descending competitive performance

In summary, our results identify a multi-synaptic forebrain-MDT-cACC circuit that regulates hierarchy formation and competitive ability in parallel with the previously described MDT-dmPFC pathway. We uncover how molecular, physiological, and circuit-level mechanisms affecting cortical excitatory-inhibitory balance control state-dependent behavior and regulate social interactions.

### Limitations of the study

Our study identifies MDT and cACC’s role in modulating competitive performance in stable hierarchies, and MDT’s role in hierarchy establishment; however, cACC’s function during hierarchy formation remains unexplored. We demonstrate that MDT—>cACC projections inhibit cACC^Pyr^ activity and promotes winning—opposite to dmPFC, where winners display increased pyramidal activity. This suggests that these pathways work synergistically in competitive outcomes, though precise mechanisms require further investigation. While we identify TRPM3 as a regulator of competitive performance and MDT^Glut^ neuronal physiology, we have not determined whether pre-existing *Trpm3* expression variability influences initial rank establishment. Finally, though we show that *Trpm3* is differentially expressed by rank and contributes to MDT^Glut^ activity, other ion channels may also play significant roles in social rank-dependent MDT^Glut^ neuronal function.

## Resource Availability

### Lead contact

Further information and requests for resources and reagents should be directed to and will be fulfilled by the lead contact, Catherine Dulac (dulac@fas.harvard.edu).

### Materials availability

This study did not generate new unique reagents.

### Data and code availability

Single nucleus sequencing transcriptomic data have been deposited at NCBI GEO [accession number: GSE300694]. This paper used existing R and Matlab code packages and does not report original code. Any additional information required to reanalyze the data reported in this paper is available from the lead contact upon request.

## STAR METHODS

### EXPERIMENTAL MODEL AND STUDY PARTICIPANT DETAILS

#### Experimental animals

Five mouse lines were used. C57BL/6J were shipped as littermates from Jackson laboratory. Vglut2-ires-Cre ^68^ (B. Lowell, Harvard Medical School) PV-IRES-Cre ^69^ (S. Arber, Friedrich Miescher Institute; provided by N. Uchida, Harvard University) and VGAT-Cre (JAX #:028862) mice were imported and bred in a barrier facility at Harvard University. PV-reporter mice (PV-IRES-Cre; Ai14-lsl-tdTomato) were a provided by T. Hensch (Harvard University). Animals were weaned at three weeks of age and maintained on a 12:12 hr light:dark cycle (lighted hours 02:00 to 14:00) with food and water ad libitum. Animal care and experiments were carried out in accordance with the NIH guidelines and approved by the Harvard University Institutional Animal Care and Use Committee (IACUC). All assays were performed on adult male mice (age > eight weeks) unless otherwise noted.

### METHOD DETAILS

#### Behavioral Assays

##### Social hierarchy paradigm and Elo ratings.

Hierarchies were established in groups of three or four mice. Groups of four mice were used in assays testing hierarchy dynamics, lesions, and chemogenetic manipulations of MDT^Glut^ and cACC^PV^. Groups of three mice were used to investigate MDT→cACC, OFC→MDT, and BF→MDT projection neurons, TRPM3 manipulations, and all electrophysiological recordings in MDT. Groups were established by randomly picking one mouse from separate litters and matched for age (within two weeks) and weight (on average within 2 grams) and individually housed for an average of five days before experiments began. Single housing promotes territorial behavior and allowed us to control for litter effects (e.g., maternal effects) and prior experiences among group members. Mice remained individually housed for the duration of all experiments. Mice competed in tournaments of resident-intruder interactions and tube tests ^70^. Each tournament was comprised of two round robins: the first consisted of each pairwise combination of animals, and the second consisted of the same pairwise combinations but with reversed ordering of the individuals (Fig. 1A). The group was first subjected to a tournament of resident-intruder (RI) interactions under dim red lighting. In groups of four, each tournament consisted of 12 interactions, where each mouse served as the resident and intruder in every unique pairing within a group of four mice. Similarly, in groups of three mice, each tournament consisted of nine interactions. Each RI interaction lasted 30 minutes to 1 hour. The full tournament took on average six days. Next, the group was subjected to tournaments of the tube-test ^19,71^ under dim white light from floor lamps. Tournaments were repeated for at least seven days. Wins, losses, and time to decision were recorded.

Elo rating continuously updates player rating based on whether performance is better or worse than expected from previous contests; expected outcomes lead to smaller point changes than unexpected outcomes (Methods). Elo ratings were determined with the EloRating R package ^23^. Hierarchy stability was determined by applying the EloRating stability function as a moving average across each round robin. A stability of one indicates no rank changes—whereas a stability of zero indicates rank changes for all four animals—from one round robin to the next. Hierarchy consistency was determined for each unique pair of animals in a group and is a measurement of whether the outcome in one interaction is consistent with the next interaction. A sliding window of four interactions in groups of four (or three interactions in groups of three) determined consistency, where the count was reset to zero each time the outcome between a pair changed and reached a maximum of four (or three) for each window. Individual performance was determined with “delta Elo,” or the change in Elo from one round robin to the next.

For hierarchies receiving experimental manipulations (odor swab, MDT lesion, or CNO injection), manipulations were delivered only after the hierarchy reached stability criteria. Specifically, the hierarchy had to have a stability of one for at least four round robins before the manipulation was made. For CNO injections during hierarchy interactions, animals were habituated to mock intra-peritoneal (i.p.) injections prior to tests.

##### Defensive and aggressive behavior.

Resident intruder interactions were video recorded in parallel using a multi-camera surveillance system (Geovision GV-1480 capture card) and CCD cameras. We defined defensive behavior as fleeing or avoiding the other mouse, including curling up in a C shape when being pursued or standing up on two feet and pushing away a pursuer mouse. Defensive frequency, duration, rate, and index (defined as the fraction of frequency and duration measurements of a given mouse divided by the total of the pairwise interaction) were recorded using Observer XT 8 software (Noldus Information Technology). To address how predictive agonistic behaviors were of eventual tube rank, we used regression analysis to calculate the percent of variance in tube rank that was explained by the relative duration of aggressive and defensive behavior during resident intruder.

##### Odor transfer experiments.

Urine was collected by holding the mouse over a clean plexiglass sheet and urine was pipetted into a microfuge tube. Saliva was collected by pilocarpin injections under general anesthesia. Mice were anesthetized with 100 mg/kg ketamine (KetaVed, Vedco) and 10 mg/kg xylazine (AnaSed) via i.p. injection, and then i.p. injected with 0.5 mg/kg pilocarpine. Saliva was collected by pipetting directly from the mouth into a microfuge tube. In transfer experiments urine and saliva were either used fresh or frozen at −80°C and thawed immediately prior to the experiment. During odor transfer experiments, all mice in a social group were lightly anesthetized using isoflurane (VetOne), washed with soap and water, and padded down with 70% ethanol. Urine, saliva, or both was painted on to the face and neck with a paintbrush (< 50 μL per substance). The painted animal was allowed to habituate to the swabbed odors for at least 10 minutes before beginning round robin tube test tournaments.

##### Sociability and declarative social memory assay.

The nonconditioned social discrimination procedure was performed under dim red light essentially as described ^72^. Mice were habituated to the three-chamber apparatus (25.5 × 36 cm) consisting of two side chambers and a connecting corridor on multiple days before the test date. Sociability was measured during the learning phase of the experiment, where a juvenile (aged 16–35 days) was positioned under an inverted wire pencil cup in one side chamber and an empty inverted wire pencil cup was placed in the other chamber; the adult test animal was allowed to explore the apparatus for seven minutes. After seven minutes, test mice were given a 90-minute inter-trial intermission period in their home cage. Next, declarative memory was tested for 10 minutes during the recall phase, where the familiar juvenile from the learning phase was positioned under the same inverted pencil cup, and a novel juvenile was positioned on the opposite side under the pencil cup. The time spent in each of the three chambers was then measured and analyzed using Ethovision XT 8 software (Noldus Information Technology). In DREADD experiments, CNO or vehicle was delivered 30 minutes prior to the onset of the sociability assay.

##### Open field assay.

Open field assay was used to measure locomotion and time spent in an exposed area of a cage. Mice were placed into a large cage (25.5 × 36 cm) with a clear acrylic lid for 15 minutes. Distance traveled, velocity, and time spent in the center of the cage (12 × 18 cm) was measured with Ethovision XT 8 software (Noldus Information Technology).

#### On-off schedule.

The on-off schedule was used to deliver manipulations to targeted individuals during tube test tournaments. During “on” tournaments, one of the animals received the manipulation (CNO, agonist/antagonist, or blue/red light), while the remaining animals in the group either received the vehicle (saline) or were not subjected to the manipulation. During the intervening “off” tournaments all mice were untreated.

#### Physiological measurements

Sperm and salivary glands were recovered immediately following sacrifice in mice that had established hierarchy.

##### Sperm count.

*Cauda epydidymus* and *vas deferens* were bilaterally dissected and placed into 150 μL of PBS in a petri dish. For 12 minutes sperm were allowed to swim out, after which both organs were gently squeezed with forceps to press the remaining sperm out. All floating sperm were then collected by pipette and transferred into a microcentrifuge tube. To count sperm, 10 μL of the 150 μL suspension was mixed with 10 μL of Trypan Blue, and cell numbers determined with the Countess Automated Cell Counter (Thermo Fisher Scientific, C10227).

##### Salivary gland mass.

Salivary glands, including both submaxillary and sublingual glands but not preputial glands, were bilaterally dissected as described ^73^ and individually weighed.

#### Histology

All histological sections were mounted onto Superfrost slides (VWR, 48311–703) with DAPI-Vectashield (Vector Labs, H-1200). Sections were imaged at 10x resolution with an AxioScan Z1 slide scanner (Zeiss) or at 20x resolution in confocal stacks with an LSM 880 with Airyscan (Zeiss).

##### RNA in situ hybridization.

In situ hybridization of *Fos, Arc*, *Slc17a6* was performed as described ^74^. Fresh brain tissues were collected from animals in their home cage or 35 minutes after the mid-point of a tube test tournament, embedded in OCT (Tissue-Tek, 4583), and slowly frozen with dry ice. Whole brain, 20 μM coronal sections were kept at −80°C. Complementary DNA (cDNA) was cloned into approximately 800-base-pair segments into pCRII-TOPO vector (Thermo Fisher, K465040). Antisense complementary RNA probes were synthesized with T7 (Promega, P2075) or Sp6 polymerases (Promega, P1085) and labeled with Digoxigenin (DIG, Roche) or Fluorescein (FITC, Roche). Probes were hybridized at 68°C at a concentration of 0.5 to 1.0 ng/mL. Horseradish peroxidase-conjugated antibodies against DIG (anti-DIG-POD, Roche, 1:500) and FITC (anti-FITC-POD, Roche, 1:250) incubated overnight and developed with the TSA-plus Cy3 system (Perkin Elmer). Signals were amplified with biotin-conjugated tyramide (Perkin Elmer) and developed with Alexa Fluor 488-conjugated streptavidin (Thermo Fisher, S11223). Washes were done with 0.5% PBST (0.5% Triton X-100 in PBS).

RNAscope in situ hybridization was performed by collecting frozen coronal sections (16 μM) as described above. Hybridizations were performed using the RNAscope Multiplex Fluorescent Assay (ACDBio) according to the manufacturer’s instructions. ACDBio probes used were *Necab1* (#428541-C2), *Scn3a* (#502641), *Trpm3* (#459911) and *Fos* (506921-C2, 506931-C2). Probes were visualized with ACDBio AMP 4 Alt B fluorescent dyes (C1 = Atto 550; C2 = Atto 550, C3 = Atto 647). Images were acquired on a Zeiss Axioscan microscope.

##### Fluorescence immunohistochemistry.

Staining with FOS, NeuN, GFAP and PV antibodies was performed according to standard protocols. Animals were perfused with phosphate buffered saline (PBS) followed by 4% paraformaldehyde (PFA). Dissected brain tissue was post-fixed in 4% PFA overnight and washed and stored in PBS. Whole brain, 60 μm sections were collected by embedding the brain in 4% low melting point agarose (Thermo Fisher Scientific, 16520–050) and sliced on a Vibratome (Leica). Sections were made permeable and blocked overnight against nonspecific fluorescence with blocking buffer (0.5% Triton X-100, 1% BSA, 2% normal donkey serum in PBS). Primary and secondary antibody incubations were in blocking buffer on a rotator at 6 hours room temperature or overnight at 4°C. Primary and secondary antibodies were washed with 0.5% PBST (5 × 60 min). Primary antibodies: rabbit anti-FOS 1:2500 (Synaptic Systems 226003); mouse anti-NeuN 1:500 (Chemicon A60); rabbit anti-GFAP 1:000 (Abcam, Ab7260); rabbit anti-parvalbumin 1:1000 (Swant, PV27). Secondary antibodies (all from Thermo Fisher): Alexa-568 anti-goat (A-11057) 1:1,500, Alexa-555 anti-goat (A-21432) 1:1,500, and Alexa-647 anti-goat (A-21447) 1:1,500. FOS antibody signal was amplified by incubation with Biotinylated horse anti-rabbit (Vector Laboratories, BA-1100) at 6 hours room temperature or overnight at 4°C and developed with Streptavidin, Alexa Fluor 555 Conjugate (Life Technologies, S32355); both steps were washed in PBST (5 × 60 min).

#### Stereotaxic Surgeries

All surgeries were performed under aseptic conditions. Animals were anesthetized with 100 mg/kg ketamine (KetaVed, Vedco) and 10 mg/kg xylazine (AnaSed) via intra-peritoneal (i.p.) injection. Viruses and NMDA were stereotactically delivered with Nanoject II injector (Drummond Scientific) at a final volume of 135 nL per injection site. Analgesia (buprenorphine, 0.1 mg/kg, i.p.) was administered for 2 days following each surgery.

##### Guide cannulas for drug delivery.

Anesthetized mice were bilaterally implanted with a guide cannula (26 gauge, PlasticOne) sitting 0.5 mm above the target area, along with a dummy place holder (C235DCS-5/SPC, 0.008”) with a 0.2mm projection, and an internal (C235IS-5/SPC) with a 1mm projection beyond the guide cannula tubing. For the MDT (AP: −1.35, ML: 0.43, DV: −3.2) we used a guide cannula (C235GS-5–0.8/SPC) cut 2.95mm below the pedestal, For the cACC, (AP: +1.18, ML: 0.17, DV: −1.3 to −0.95) we used a guide cannula (C235GS-5–0.6/SP) cut 1.0mm below the pedestal. At least three weeks after cannulation, mice went under a brief isoflurane anesthesia (2–5%, 1 L/min oxygen) and mounted on a stereotaxic instrument to ensure a stable flow of drug without interruption. Drug was administered through the cannula internal at a rate of 1ul/20min using a two-channel syringe pump (Fusion 200 Touch Pump) fitted with two Hamilton Syringes (Gastight 1700 Series, Model 1702 N, Syringe 80200). Syringes and tubing were filled with water, and mineral oil and drug were subsequently backfilled into the ends of the tube, ensuring no air bubbles in the system. Cannula internals were left in place for 5 min before removal. Volume of infusion was based on the previous finding that A 0.5 μL volume spreads to approximately a 1.0 mm diameter sphere whereas 2.0 μL spreads to 2.4 mm^75^. We therefore injected 0.5 uL into the target areas.

##### Optic fiber and GRIN lens implants.

All surgeries were performed under aseptic conditions with isoflurane (1–2% at 0.5–1.0L/min) as anesthesia. For optogenetic stimulation experiments, bilateral optical cannulas (DFC_400/430–0.48_4mm_GS1.0_FLT, Doric) were implanted in the MDT (AP: −1.35, ML: 0.5, DV: −3.2). Light pulses of 5ms, delivered at 5 Hz, at 1mW/mm^2^ were used to activate the projections. For fiber photometry experiments, mono fiber optic cannulas (MFC_200/250–0.66_4mm_ZF1.25_FL, Doric) were implanted in both the right cACC (AP: 1.18, ML: 0.45, DV: −1.00) and left dmPFC (AP: 2.43, ML: 0.4, DV: 1.2). For GRIN lens implantation, we used integrated GRIN lenses (1mm diameter and 4 mm height, Inscopix) that were implanted in the right cACC (AP: 1.18, ML: 0.65, DV: −1.0).

#### Calcium imaging

##### Fiber photometry recording.

Photometry recording was done using a custom-built set-up via a branching patch cord (BBP(4)_200/230/LWMJ-0.57_m_FCM-4xMF1.25(F)_LAF, 200 μm core, Doric Lenses). After connecting the cable, animals were allowed to habituate in the cage for ~10 mins. Once the recording started, the patch cord was used to simultaneously deliver excitation light at different wavelengths (473nm and 560 nm, Luxeon Rebel Color LEDs, LedSupply). Recordings were made at 200 Hz and the emitted light was recorded with a FLIR camera (BFS-U3–16s2M-CS). The timing of the LED pulses and the frame start of the photometry camera were controlled by the frame start of the behavior camera.

##### Microendoscope and behavioral assays.

Optimal imaging settings were determined on the first day of the imaging experiments. The implanted mice were transiently restrained and the microendoscope (nVoke and nVue, Inscopix) was attached onto the lens assembly on the head of the mouse. The focusing plane of the microendoscope was carefully chosen to choose an imaging plane with most neurons. The field of view was cropped to the region encompassing the fluorescent neurons. The imaging settings were saved for individual animals and preloaded before starting the experiment on subsequent days. This allowed us to rapidly switch between the animals during a behavior assay. We used inbuilt acquisition software from Inscopix to acquire images at 10Hz. Mice were habituated to the microendoscope setup two times before the formal behavior assay. During every tube test session, we recorded the baseline activity of the mice for 1 min in their home cage followed by the tube test. A National instrument (USB-6501) controller was used to deliver a TTL signal to synchronize the behavior camera (BFS-U3– 31S4M-C, FLIR Blackfly S camera) and the two microendoscope cameras.

#### Viruses, drugs, and CTB

##### Virus titers.

AAV8-hSyn-DIO-hM3D(Gq)-mCherry (2.7*10^13^ vg/mL). AAV8-hSyn-DIO-hM4D(Gi)-mCherry (7.9*10^12^ vg/mL). AAV-DIO-TVA-mCherry (3.4*10^12^ vg/mL). AAV-DIO-RG (1.4*10^12^ vg/mL). AAV-Syn-ChrimsonR-tdT (7×10^12^ vg/mL). AAV-Syn-FLEX-ChrimsonR-tdTomato (5×10^12^ vg/mL). AAV5-Syn-Chronos-GFP (5.7*10^12^ vg/mL). AAV-DIO-TVA-mCherry (3.4*10^12^ vg/mL). AAV-DIO-RG (1.4*10^12^ vg/mL). AAV-CamKII-GCaMP6s-WPRE-SV40 (1×10^13^ vg/mL). AAV-Ef1a-Con/Foff 2.0-sRGECO (1×10^13^ vg/mL). AAV-hSyn-Cre-P2A-dTomato (7×10^12^ vg/mL). AAV-hSyn-DIO-HA-hM3D(Gq)-IRES-mCitrine (1×10^13^ vg/mL). AAV-CaMKIIa-HA-hM4D(Gi)-IRES-mCitrine (1×10^13^ vg/mL).

##### In vivo chemogenetic inhibition and excitation of MDT and cACC.

Excitatory (pAAV-hSyn-DIO-hM3D(Gq)-mCherry) and inhibitory (pAAV-hSyn-DIO-hM4D(Gi)-mCherry) Serotype 8 DREADD-expressing viruses^76^ were produced from UNC Vector Core (discontinued) and Addgene (44361 and 44362).

##### In vivo chemogenetic excitation of MTD-cACC and concurrent inhibition of dmPFC.

Each animal received cranial injections of three viruses (purchased from Addgene). A retrograde AAV encoding Cre recombinase (pAAVrg-hSyn-Cre-P2A-dTomato; 107738-AAVrg) was injected into the cACC; Cre-dependent excitatory DREADD (pAAV-hSyn-DIO-HA-hM3D(Gq)-IRES-mCitrine; 50454-AAV8) was injected into the MDT; inhibitory DREADD under the CamKII promotor (pAAV-hSyn-DIO-HA-hM3D(Gq)-IRES-mCitrine; 50467-AAV8).

##### Anterograde tracing.

Anterograde tracing was performed in adult naïve Vglut2-ires-Cre male mice. MDT coordinates: AP: −1.35, ML: 0.43, DV: −3.2. A 1:1 mixture of AAV1/CAG-FLEx-tdTomato and AAV1/CAG-FLEx-Syn-GFP ^77^ was injected to visualize presynaptic terminals of MDT^Vglut2^ neurons. The relative density of synapses was calculated as follows. ROIs corresponding to each brain region were obtained with a custom ImageJ script, and the Syn-GFP normalized mean fluorescence (i.e., raw integrated density / area in pixels) was calculated, along with the normalized mean of a dark (i.e., unlabeled) section of the same slice. With an R script we then calculated, for each ROI, the Syn-GFP normalized mean / dark normalized mean to derive the synaptic density of that region. Finally, to determine the relative synaptic density of each ROI across replicates, we divided each ROI mean density by the summed mean density of all ROIs.

##### Monosynaptic retrograde tracing.

Input tracing experiments were performed in adult naïve Vglut2-ires-Cre and PV-IRES-Cre mice (cACC coordinates: AP: +1.18, ML: 0.17, DV: −1.3 to −0.95) mice. Cre-dependent helper AAVs ^78^ containing the TVA receptor for avian virus envelope protein (EnvA) and rabies glycoprotein (AAV-DIO-TVA-mCherry, AAV-DIO-RG) were injected in a 1:1 mixture. After 10 days of expression, EnvA-pseudotyped, G-deleted rabies virus (SBPN-RbV-EnvA-GFP, Salk Vector Core) (Wickersham et al. 2007) was injected into the same coordinates. After 4 days of expression, brains were prepared for histology. We quantified the number of region-specific inputs for each mouse in two steps. First, we calculated the convergence index (i.e., the number of region-specific inputs divided by the total number of starter cells). Second, we normalized the convergence index by the percentage of starter neurons specifically located in the MDT. Thus, this metric accounts for the number of starter cells and the percentage of MDT-specific stater cells, for each mouse.

##### *In vitro* optogenetic stimulation.

Two channel-rhodopsin (ChR) variants under the human synapsin promoter (Syn) were used: ChrimsonR (AAV5-Syn-ChrimsonR-tdTomato-WPRE-bGH; V5453) and Chronos (AAV5-Syn-Chronos-GFP), both produced from the UNC Vector Core. One variant with the CamKII promoter was used (AAV-CaMKII-Chronos-GFP, serotype AAV5), produced from the Duke Vector Core.

##### *In vivo* optogenetics stimulation.

To selectively target two projection pathways in the same animal in vivo, cohorts of three mice (Vgat-Cre) received bilateral injections of conditional Chrimson (AAV-Syn-FLEX-ChrimsonR-tdTomato, 200nl) in BF and non-conditional Chronos (AAV-Syn-Chronos-GFP, 200 nl) in OFC.

##### CTB tracing.

Retrograde tracing experiments were performed in groups of C57BL/6J mice with established tube test hierarchies (among groups of three). Cholera toxin B (CTB) conjugated to Alexa Fluor-488, −555, or −647 (Thermo Fisher: CTB-488 C22841; CTB-555 C34776; CTB-647 C34778) was injected into the cACC in each mouse, as well as other areas known to receive projections from the MDT (dorsal-medial striatum, orbitofrontal cortex, agranular insular cortex, retrosplenial cortex). Five days after surgery, the mice were returned to daily tube test tournaments. Seven days after surgery, the mice were sacrificed 90 minutes after the mid-point of a tube test tournament and perfused with 4% PFA. Brains were post-fixed in 4% PFA, sectioned at 60 μm, and co-stained for FOS. The number of CTB-FOS co-labeled cells was quantified in the MDT.

##### MDT Lesions.

N-methyl-D-aspartate (NMDA) in PBS (0.25 M) was bilaterally injected into MDT at a final volume 135 nL over a 20 min period. At the end of the injection, the pipet remained at the site for 5 min to allow for diffusion of the solution. Vehicle (PBS) was injected according to the same conditions. Lesions were confirmed with NeuN immunohistochemistry.

##### Cannulated drug delivery.

To activate DREADD receptors, CNO was administered at a concentration of 0.5 ug/ul CNO solution in PBS with 0.25% DMSO. To manipulate the TRPM3 channel in the MDT, an agonist (CIM0216, Tocris) or antagonist (ononetin, Tocris 5143) was delivered at a concentration of 20μM. The stock solutions of the drugs were prepared in DMSO and ethanol for ononetin and CIM0216 respectively, at 5mM concentration and were further diluted in saline to a final concentration of 20 μM on the day of the experiments and 500 nL of the drugs was infused bilaterally over the duration of 10 minutes.

#### Imaging and image analysis

##### Histological imaging.

Samples were imaged using an Axio Scan.Z1 slide scanner (Zeiss), and confocal stacks were acquired on an LSM 880 confocal microscope (Zeiss). Image processing was performed using custom routines for the Fiji distribution of ImageJ. Cell counts were performed by bilaterally cropping out anatomical regions of interest (ROI) followed by cellular segmentation with parameters customized to color channel and anatomical ROI.

##### RNAscope.

For RNAscope puncta quantification, a custom FIJI script was used to manually crop left and right *Necab1*+ MDT brain regions; crops were separated into three separate images corresponding to DAPI (blue), *Necab1* (green), and the candidate gene (*Trpm3* or *Scn3a*) or *Fos* in red. A CellProfiler pipeline ^79^ was created to measure RNA signal of candidate genes within *Necab1*^+^ and *Necab1*^−^ cells. Nuclear boundaries (DAPI) were assigned by a global, minimum cross entropy algorithm. *Necab1* puncta were segmented using the adaptive Otsu thresholding method. Nuclear boundaries were expanded by 4 microns; expanded nuclear boundaries containing *Necab1* puncta were defined as *Necab1*+ cells, whereas expanded nuclear boundaries lacking *Necab1* puncta were defined as *Necab1*^−^ cells. To quantify puncta of candidate genes (i.e., *Trpm3* and *Scn3a*) within these two cell types, raw images were enhanced for speckle detection, segmented using the adaptive Otsu thresholding method, and puncta labeled with the RelateObjects function.

#### Electrophysiology

##### Solutions.

Modified ACSF (artificial cerebro-spinal fluid) contained (in mM): 105 choline chloride, 20 glucose, 24 NaHCO_3_, 2.5 KCl, 0.5 CaCl_2_, 8 MgSO_4_, 5 sodium ascorbate, 3 sodium pyruvate, 1.25 NaH_2_PO_4_ (osmolarity 290, pH 7.35) was used as cutting solution. All recordings were made in an oxygenated ACSF with composition (in mM): 115 NaCl, 2.5 KCl, 25 NaHCO_3_, 1.25 NaH_2_PO_4_, 1 MgSO_4_, 20 glucose, 2.0 CaCl_2_ (osmolarity 290, pH 7.35). The modified ACSF was used for isolating barium currents (in mM): 95 NaCl, 10 HEPES, 2.5 KCl, 1 MgCl_2_, 0.5 BaCl_2_, 20 glucose, 20 TEA, and 1.25 NaH_2_PO_4_, and 4 4-AP and 0.001 TTX.

Current-clamp internal solution contained (in mM): 120 potassium gluconate, 2.0 sodium gluconate, 10 HEPES, 4.0 Mg-ATP, 2.0 Na_2_-ATP, 0.3 Na_3_-GTP, and 4.0 NaCl (osmolarity 292, pH 7.34). Voltage clamp internal solution contained (in mM): 130 d-gluconic acid, 130 cesium hydroxide, 5.0 NaCl, 10 HEPES, 12 di-tris-phosphocreatine, 1 EGTA, 3.0 Mg-ATP, and 0.2 Na_3_-GTP (osmolarity 291, pH 7.35). To view cells after recording, internal solutions contained 1% biocytin or Alexa 488 or 594 as indicated. All chemicals were purchased from Sigma-Aldrich.

##### Acute brain slices.

In vitro slice physiology was performed in adult (approximately two months) C57BL/6J mice with established tube test hierarchies (in groups of three) and in age-matched, individually housed controls. Mice were sacrificed during the inactive cycle at least two days after the last tube test tournament. Slices were prepared using methods described previously ^80^. Mice were lightly anesthetized with isoflurane exposure using a vaporizer (Datex-Ohmeda) connected to a clear acrylic chamber for two min, and then deeply anesthetized with an i.p. injection of a mixture of ketamine (100 mg/kg) and xylazine (10 mg/kg). Mice were transcardially perfused with ice-cold modified ACSF cutting solution, and brains were dissected into the same solution. Sagittal slices (300 μm thick) were obtained using a vibratome (VT1000S; Leica, Germany) and collected in ice-cold cutting solution. MDT was identified based on its proximity to anatomical landmarks including the fimbria, third ventricle, and mammillothlamic tract. cACC^PV^ neurons proximal to the genu of corpus callosum were identified by the tdTomato reporter. After cutting, slices were incubated in oxygenated ACSF solution at 35 °C for 45 min and then at room temperature for the duration of the experiment.

##### *Ex vivo* recordings.

Whole-cell current-clamp and voltage-clamp recordings were made using borosilicate glass patch pipettes (6–10 Mohm) filled with current-clamp internal and voltage-clamp internal solutions, respectively, and slices maintained at 35 °C in oxygenated ACSF. Slices were visualized under custom-built infrared optics on a BX51WI microscope (Olympus Optical, Tokyo, Japan). Recordings were obtained with a Multiclamp 700B amplifier (Molecular Devices, Palo Alto, CA), and physiological data were collected via software written in LabView (National Instruments) and pClamp 10.3 (Molecular Devices). After breaking into the cell and throughout the recording session multiple input resistance measurements were made under the voltage clamp conditions using a very small (10 mV hyperpolarizing) and a brief (20 ms) pulse. To visualize recorded cells, slices were fixed in 4% PFA overnight, washed with PBS, incubated in blocking buffer (0.3% Triton X-100, 2% normal goat serum in PBS) for four hours, stained overnight in blocking buffer with Streptavidin Alexa Fluor 594 or 647 conjugate (Life Technologies S11227) at 4 °C, and washed in 0.5% PBST. All neurons were multipolar as previously described in rodent MDT ^81^.

For optogenetic axon terminal photostimulation experiments, Chronos or ChimsonR were activated using custom-built 465 nm (CBT-90-B-L11, Luminus) or 625 nm LEDs (CBT-90-RX-L15, Luminus). To record synaptic input to MDT cells, ChRs were transduced into cells of the following areas: orbitofrontal cortex (OFC), piriform cortex, lateral preoptic area, ventral pallidum, olfactory tubercle, and the diagonal band nucleus (HDB). Light pulses of 5ms, delivered at 1–10 Hz, were used to evoke synaptic transmission.

##### Short term plasticity “paired pulse” measurements.

Short term plasticity of inputs to MDT neurons was measured by measuring eEPSC amplitude of five successive evoked responses (10 ms light stimulus) separated by a 100 ms interval. Effects of social rank on the probability of release (e.g., facilitation or depression) were determined by dividing the current amplitude of each evoked response (i.e. first through fifth) by the amplitude of the first response.

##### White noise experiments.

We generated a “white noise” paradigm that evokes a reliable and stable firing pattern (Ermentrout et al., 2008; Galán et al., 2008), thus providing a standardized physiological environment on which to determine the effect of MDT^Glut^ input manipulations. We created 400 random patterns of noisy input (standard deviation sigma= 50 pA) and applied a low pass filter by convolving these with an alpha function (alpha= 2ms), and randomly selected 200 of them to inject into cACC^Pyr^ neurons to generate a simulated peristimulus time histogram of neuronal firing. We activated the MDT fibers by shining 5 pulses of blue light (5ms at 10 Hz of 465nm).

##### Synaptic activity measurements.

Synaptic data was analyzed in Axograph using a template match algorithm. A low pass filter (2 kHz) and a notch filter (60 Hz and its harmonics) were applied to the imported data to reduce the noise. Parameters for excitatory event detection: variable amplitude, rise time of 0.5 ms and decay time of 2 ms, baseline length of 3 ms, and template length of 7 ms. Parameters for inhibitory event detection: variable amplitude, rise time of 1 ms and decay time of 3 ms, baseline length of 5 ms, and template length of 10 ms. The threshold for detection of both the inhibitory and excitatory events was set to be 3* standard deviation of the baseline noise.

##### TRPM3 pharmacology and barium current recordings.

For pharmacological manipulation of TRPM3 channels, the agonist CIM 0216 (Tocris, 5521) and antagonists mefenamic acid (Sigma, 92574) and ononetin (Tocris) were added separately to the recording solution. MDT membrane voltage responses to the drugs were recorded in current clamp mode.

Barium currents were recorded as a proxy for calcium currents, to get better signal-to-noise ratio, and to minimize inactivation ^49,50^. Barium currents were recorded in the presence of compounds to block sodium and potassium currents in modified oxygenated ACSF with composition (in mM): 95 NaCl, 2.5 KCl, 10 HEPES, 1.25 NaH_2_PO_4_, 1 MgCl_2_, 20 glucose, 0.5 BaCl_2_, 4 4-AP, 20 TEA and 1μm TTX (osmolarity 290, pH 7.35). Neurons were held −70 mV, hyperpolarized to −110 mV, and then depolarized in 5 mV increments to 30 mV.

To determine the TRPM3 component of the barium current, we used the following equation: [peak control current - peak current in presence of TRPM3 antagonist] / peak control current], where control current refers to the barium current.

##### Drugs.

4-AP (Tocris, 0940); TTX (Hellobio, HB1034); CIM 0216 (selective TRPM3 channel agonist; Tocris, 5521); Mefenamic acid (nonselective TRPM3 antagonist; Sigma, 92574); Ononetin, (selective TPM3 antagonist; Tocris 5143).

#### Single nucleus RNA-Seq

##### Nuclear isolation, purification, and barcoding.

Nuclei were extracted following a custom protocol designed to minimize the amount of time from euthanasia to nuclear barcoding (approximately four hours). We prepared 10 biological samples for sequencing. Each biological sample was a pool of three mice to obtain sufficient material. Nine of the biological samples came from three hierarchy cohorts, each cohort consisting of rank-1, rank-3, and control pools. The tenth biological sample was an additional control pool. Thus, the 10 biological samples were comprised of 30 animals. All animals were age-matched and processed in parallel. Mouse brains were quickly dissected on ice and 2mm thick coronal sections were cut on a brain matrix (Zivic Instruments, #5325). Sections were placed on a chilled surface under a dissecting microscope and tissue punches from the thalamus were collected using a 2mm diameter cylindrical corer (Fine Science Tools, #18035–02). Tissue punches were pooled in chilled Hibernate-A Medium (ThermoFisher, #A1247501), minced into approximately 0.5^2^ mm pieces, and transferred to 3 mL lysis buffer (10 mM Tris-HCl, 10 mM NaCl, 3 mM MgCl2, 0.1% Nonidet P-40). Tissue was dounce homogenized in a glass tube 6–10 times with a grinder pestle (Potter-Elvehjem, #886000–0020) rotating at 300 rpm until opaque, transferred to a 15 mL tube, and pelleted in a swinging bucket centrifuge (Beckman Coulter GS-6R) at 50 RCF for 5 min at 4°C. Supernatant was removed and tissue carefully resuspended in 1.5 mL PBS with BSA (1%) and RNase inhibitor (0.2 U/μL, Promega, #N2615). To remove debris, the suspension was passed over a 70 μM filter (ThermoFisher, #NC0120755) and then again over a 20 μM filter (ThermoFisher, #130–101-812). With the suspension on ice, nuclei were labeled by adding DAPI (1.5 μL, ThermoFisher, #D1306). DAPI-positive nuclei were sorted at 4°C on a FACSAria flow sorter (BD Immunocytometry Systems) with a 70 mm nozzle on the four-way high speed purity setting until approximately 100,000 nuclei were collected.

Finally, to estimate nuclear concentration, 10 uL of suspension was transferred to an Eppendorf tube and mixed with 1 μL propidium iodide (ThermoFisher, #P21493), counted on a Luna automated cell counter slide (Logos Biosystems), and adjusted to a final concentration of approximately 500 nuclei per microliter for 10X GEM formation. In total, 10 pools of nuclei were sequenced, where each pool consisted of thalamic microdissections from two mice (N = 20 mice). Nuclear pools were made from dominant (rank 1; 3 pools), subordinate (rank 3; 3 pools), and individually housed controls (4 pools). We profiled 33,582 neurons from rank-1, rank-3, and control mice.

##### Single nucleus library preparation and RNA-Seq.

Using the 10X Genomics droplet microfluidics Gemcode platform ^83^, nuclei from the suspension were loaded onto Chromium instrument. Libraries were generated using Chromium v2 reagents, and cDNA amplified according to the manufacturer’s instructions. Samples were sequenced on an Illumina Nextseq at the Harvard University Bauer Core Facility. We recovered an average of 4,699 transcripts and 2,169 unique genes per nucleus.

##### Sequence alignment and identification of variable genes.

Transcript sequences were demultiplexed, aligned to the mm10 mouse genome, counted, and assembled into gene-cell matrices using the 10x Genomics Cell Ranger pipeline with default parameters. The matrices were then analyzed in Seurat version 2.3.4 ^84^. Gene expression for each nucleus was log-normalized and scaled (i.e., log-transformed, normalized by total transcript count, and multiplied by a factor of 10,000). To eliminate aberrant droplets, we filtered out nuclei based on mitochondrial content (> 10%), nuclear doublets (> 20,000 UMIs), red blood cells (>1% hemoglobin genes), and immediate early gene (IEG) responses (e.g., responses to the dissection/dissociation process; > 1% IEG content). Glial cells were filtered out based on the expression of established glial-specific genes. To identify variable genes that characterize cellular heterogeneity based on dispersion (variance/mean) in expression level, we used the FindVariableGenes function with the following parameters (x.low.cutoff = 0.1, y.cutoff = 0.8). To control for unwanted variation in nuclear expression profiles of the variable genes, we used regression to control for the effects of nuclear UMI count, percent mitochondria, percent IEG, and batch identity using the ScaleData function with ‘negbinom’ for the model parameter; the residual matrix was then scaled and centered and used for downstream analysis.

##### Dimensionality reduction, clustering, and marker gene identification.

Dimensionality reduction was performed with principal components analysis (PCA) of the variable gene residual matrices as implemented in Seurat ^39^. To determine the number of principal components (PCs) to use in downstream analyses, we used the JackStraw function to compare PCA scores of random subsets of genes with the observed data, allowing us to find significant PCs with a strong enrichment of genes with low P-values. We also used the PCElbowPlot function to visualize the cumulative standard deviations of each PC and the point at which successive PCs explained a diminishing degree of variance.

We defined cell clusters by passing the first 45 PCs to a shared nearest neighbor (SNN) clustering algorithm as implemented in the FindClusters function; for this analysis we used the smart local moving (SLM) community detection algorithm and a K-nearest neighbor value of 19. To visualize nuclei according to their PC scores, we used the UMAP algorithm to place each nucleus on a two-dimensional plot; subsequently, each nucleus was colored based on cluster identity.

To nominate cluster-specific marker genes based on differential expression, we used the FindAllMarker function with the MAST algorithm ^85^; differentially expressed genes that were expressed in at least 15% of cells within the cluster and with a fold change of more than 0.5 (log scale) were selected as markers. To determine whether clusters corresponded to previously-uncharacterized or well-established cell types (e.g. inhibitory, excitatory, oligodendrocytes), we examined the expression patterns in the mouse brain using the Allen Brain Atlas ^86^.

##### Thalamic sNuc-Seq and validation.

We identified a distinct cluster corresponding to the reticular thalamus, a known inhibitory (GABAergic) population ^87^ that robustly expressed *Gad1*, *Gad2,* and the new marker *Isl1*. The majority of sequenced nuclei (20,533, 61%) belonged to one large metacluster comprised of 13 clusters, each identified to be excitatory thalamic areas based on expression of *Vglut1* and *Vglut2* (SOM Fig. 6E) ^86^. Nine of these 13 clusters were assigned to paraventricular (associated with *Zbtb20*, *Dlgap2*), dorsal (associated with *Hcn1* and *Kirrel3*), and ventral (associated with *Galnt18*) thalamic regions, and the remaining five assigned to the MDT. The five MDT clusters were organized within two nodes on the dendrogram corresponding to three *Kcnip3* MDT-body clusters and two *Necab1*-associated MDT-ring clusters. These clusters were characterized by 12 genes as follows: cluster 6 ring (*Myo1b*, *Rassf3*, *Sox5os4)*; cluster 15 ring (*Col12a1*, *Ror1*, *Syt17*); cluster 17 body (Adamts19, Car4, Eps8l2); cluster 19 ring (*Oprm1*); cluster 20 body (*Cnksr3*, *Gbe1*).

### QUANTIFICATION AND STATISTICAL ANALYSIS

We used linear models (lm), linear mixed-effect regression (lmer) models^88^, generalized linear models (glm), Wilcoxon-rank sum tests, and Kolmogorov–Smirnov tests (KS). Where appropriate, data were transformed to achieve more normal distributions. *Post hoc* analyses were done using ANOVA, Tukey’s honest significant difference (HSD) test, Dunn’s multiple comparisons, or least-square means contrasts. A value of p ≤ 0.05 was considered significant. All analyses were done in R and Matlab.

#### Behavioral assays.

To determine differences in hierarchy stability and pairwise consistency between observed and random data (generated by selecting a random outcome for an equivalent number of trials per group for that of the observed data), we used lmer. Stability and consistency were the response variables and explanatory variables were condition (observed vs. random), tube rank, and social group as a random effect.

To investigate the strength of association between resident-intruder defensive behaviors (i.e., defensive duration or defensive index) and tube rank, we used Akaike information criterion (AIC) selection of three nested models, each with tube rank as the response variable. In the full model, explanatory variables were defensive duration, defensive index, and social group as a random effect; in the second model, defensive duration was excluded; in the third model, defensive index was excluded. To determine the probability of defensive behavior based on tube rank, we used a binomial logistic regression by modeling defensiveness (yes or no) as the response variable and explanatory variables included simplified tube rank (upper rank vs. lower rank) and interaction time.

To investigate the role of the MDT ablation in hierarchy dynamics, we first used a sliding window to measure hierarchy stability and pairwise consistency on a per-round-robin basis. We next used lmer by modeling stability or consistency as the response variable and explanatory variables were condition (lesion or sham) and social group as a random effect. Pairwise contrasts were determined by Dunn’s multiple comparison test. To determine the effect of MDT ablation on decision timing in the tube test, we used a lmer with decision time as the response variable, and explanatory variables were condition and tournament number.

To investigate the role of the MDT in pheromonal regulation of social hierarchy, we determined the performance of rank4 males when swabbed with rank1 urine in the lesion and sham groups. A lmer was used to model delta Elo as the response variable and explanatory variables included swab condition (untreated, swab, or washed as a swab control), tube rank, the interaction between swab condition and tube rank, and social group as a random effect. Post hoc comparisons were made using least-square means contrasts.

#### Physiological measurements.

To investigate the association between tube rank and testosterone-dependent traits we used lmer by modeling sperm count or salivary gland weight as the response variable and explanatory variables included tube rank, body mass, and social group as a random effect.

#### Histology, tracing, and cellular image analysis.

To investigate the association between brain-wide neural activity and tube rank, we used lmer by modeling the percentage of *Fos*-positive cells as the response variable, and explanatory variables included tube rank and social group as a random effect. We first used the two-sided t-statistic to investigate correlation matrices of *Fos* expression between each brain region in rank1 and rank4 males. Next, to determine the effect of rank on brain-wide *Fos* correlations, we evaluated the difference between the rank1 and rank4 t-statistic matrices.

To investigate the effect of tube rank on the activity of MDT-cACC projection neurons, a lmer was used to model the number of FOS-CTB co-labeled cells as the response variable, and explanatory variables included tube rank and social group as a random effect.

To investigate the effect of tube rank on the number of puncta within genetically define cell types, a LMER was used to test the number of puncta per cell type as the response variable; explanatory variables included cell area and tube rank as fixed effects, and social group and histological slice ID were included as random effects.

#### In vivo chemogenetic inhibition and excitation of MDT and cACC.

To investigate the effect of chemogenetic neuronal manipulation on individual performance we modeled change in Elo as the response variable and explanatory variables were CNO condition (untreated, saline, or CNO), tube rank, the interaction between CNO condition and tube rank, and social group as a random effect. To determine the significance of CNO treatment on individual performance, *post hoc* comparisons were made using least-square means contrasts.

#### Electrophysiology recordings.

Whole cell patch clamp data were analyzed as previously described ^89^. To investigate the boundary between two clusters of cells based on membrane resistance we used K-means clustering. Wilcoxon rank-sum tests were used to compare the distributions of cluster-1 and cluster-2, and to test the effect of rank on the frequency and size of EPSCs and IPSCs. We used non-parametric two sample Kolmogorov-Smirnov test (KS test) to calculate the significance of mefenamic acid on firing rate of MDT neurons.

To compare the paired pulse responses across animals we used Wilcoxon ranksum test. We also used Wilcoxon ranksum test to compare the amplitudes of minimum and maximum evoked EPSCs and IPSCs. Similarly, ranksum test was used to compare the number of connections between OFC and MDT neurons, and basal forebrain and MDT neurons across different ranks.

#### Cell type specific differential gene expression and gene set enrichment.

Nuclei were classified according to their condition (rank-1, rank-3, or control). Differentially expressed genes (DEG) between each pairwise comparison (rank-1 vs. control; rank-2 vs. control; rank-1 vs. rank-3) on a per-cluster basis were identified with the Seurat function FindAllMarkers using the MAST algorithm. For each cluster, only genes expressed in at least 5% of nuclei were considered.

To determine the effect of social status on gene set enrichment, we used gene set enrichment analysis (GSEA), a technique designed to detect modest but coordinated changes in the expression of groups of functionally related genes that are defined *a priori* ^42,43^. We generated a custom list of 145 gene sets pertaining to neural structure and function (Appendix 1). Gene sets were derived from four sources as described previously ^90^ with some modifications. (1) The IUPHAR Database ^91^, a curated list of genes encoding biological targets of small molecules and licensed drugs. (2) The PANTHER Mus musculus database ^92^, a database of gene families and their functionally related subfamilies used to classify gene product function. (3) Child terms under the Gene Ontology (GO; version 2018) parent term “Synapse,” (GO:0045202). (4) Child terms under the parent term “Sequence-specific DNA binding transcription factor activity” (GO: 0003700). To be included in GSEA, each gene set had to have between 15 and 500 genes.

Enrichment score was calculated from the signal-to-noise ratio of each gene for each pairwise comparison of log-normalized gene counts, and this metric was weighted to ensure that low expression and low variance genes did not contribute to a positive enrichment score. We then performed a permutation test for false discovery rate (FDR) by permuting condition assignments 1,000 times. Gene sets with an FDR < 0.25 and an adjusted P-value < 0.01 were considered significant. Leading-edge genes were defined as those genes appearing in the ranked list before the point at which the running sum recaches its maximum deviation from zero.

To examine the intersection of GSEA and DEG datasets, we used a union of GSEA leading edge genes with differentially expressed genes exhibiting a log-fold change of at least 0.1 on a per cluster, per pairwise comparison basis.

#### Microendoscope calcium imaging.

For the inscopix data, the image processing was done in two separate software environments. First, we processed the raw image data using the Inscopix data processing software (IDPS 1.6.0.3225). The images were imported in the proprietary Inscopix format into IDPS. The images were spatially and temporally down-sampled by a factor of two to reduce the file sizes for subsequent steps without losing quality, followed by a spatial bandpass filter in the frequency band of 0.005 to 0.5/pixel. Then images were further processed using the inbuilt motion correction tool in IDPS. Calcium traces and cell identity were extracted using the inbuilt constrained non-negative matrix factorization for microendoscopic imaging (CNMFe) implementation in IDPS. The extracted traces were then imported into MATLAB environment for further analysis. Next by aligning the behavior data with the calcium imaging data from a tube test trial, we determined the timing of the first encounter between the pair of animals. We quantified the average activity of all neuronal ROIs (20 secs before and 20 seconds after the first encounter) to calculate the pre and post interaction activity.

To determine the effect of social rank on cellular calcium activity, we next calculated the distribution of differences between the post and pre activity and split the data into three clusters based upon the following criterion: neurons that deviated more than two standard deviations from zero were clustered as activated (positive change) or inhibited (negative change) and the remaining cells were classified as unchanged cells (SOM Fig. 6D). We used Wilcoxon rank sum test to compare the distributions of the three categories of neuronal ROIs according to social rank.

#### Fiber photometric calcium imaging.

For the photometry data, the data was down sampled to 20 Hz and aligned to the behavior data. We used the 30 seconds of data before the animals entered the tube as the baseline activity to quantify the changes upon entering the tube. The total duration of the time spent by animals was divided into three distinct epochs: **1**. Approach, first 1/3^rd^ of the tube. **2**. Interact, middle third of the tube. **3**. Retreat, last third of the tube. In the majority of the trials, animals encountered each other in the middle 1/3^rd^ of the tube during the tube tests. A few trials where the first interaction occurred outside the middle 1/3^rd^ of the tube, were not included in the analysis. To determine the effect of social rank on photometric calcium activity we used Wilcoxon rank sum test to compare the activity of the three epochs to the baseline activity.

To determine the precise timing of activity, including deviations in baseline activity, we first smoothened the data using Gaussian filter in Matlab. Next, after subtracting the mean of the baseline, we calculated the differential of the fluorescence data to identify the first deviation from the baseline with the criterion that five successive data points showed the deviation in the same direction and the values were at least 3 times larger than the standard deviation of the baseline. Next, we calculated the average of four successive trials to get the value of deviation for a specific rank dependent interaction. Finally, we plotted this deviation relative to the time of meeting in the middle of the tube.

## Supplementary Material

1

2

3

4

## Figures and Tables

**Table.** Stereotaxic coordinates used for implants and virus and CTB injections.

	Bregma	M/L	D/V (from top of brain)
MDT	−1.35	+/− 0/43	−3.2
cACC	1.18	+/− 0.17	−1.4 then go to −0.95
OFC	2.34	+/− 1.2	−2.1 to −2.15
BF	0.02	+/− 1.13	−4.5 to −5.25
PIR	0.62	+/− 2.61	− 4.55 to −3.9
LPOA	0.50	0.50	−4.88 to −4.7

Units: mm

**Key Resources Table:** 

Reagent or Resource	Source	Identifier
**Antibodies**		
anti-FOS	Synaptic Systems	226003
anti-NeuN	Chemicon	A60
anti-GFAP	Abcam	Ab7260
anti-parvalbumin	Swant	PV27
Probe: Necab1	ACDBio	#428541-C2
Probe: Scn3a	ACDBio	#502641
Probe: Trpm3	ACDBio	#459911
Probe: Fos	ACDBio	#506921-C2, 506931-C2
**Bacterial and Virus strains**		
pAAV8-hSyn-DIO-hM3D(Gq)-mCherry	UNC Vector Core & Addgene	Addgene: 44361
pAAV8-hSyn-DIO-hM4D(Gi)-mCherry	UNC Vector Core & Addgene	Addgene: 44362
AAV1/CAG-FLEx-tdTomato	UPenn Vector Core	# AV-1-ALL864
AAV1/CAG-FLEx-Syn-GFP	Custom; Silvia Arber (Friedrich Miescher Institute, Basel); UNC vector Core	N/A
AAV-DIO-TVA-mCherry	Salk Vector Cor	170519
AAV-DIO-RG	Salk Vector Core	N/A
SBPN-RbV-EnvA-GFP	Salk Vector Core	N/A
pAAV-Syn-ChrimsonR-tdT	Addgene	59171-AAV5
pAAV-Syn-FLEX-ChrimsonR-tdTomato	Addgene	62723-AAV5
AAV5-Syn-Chronos-GFP	UNC Vector Core	Syn-Chronos-GFP
AAV-CaMKII-Chronos-GFP	Duke Vector core	N/A
AAV-DIO-TVA-mCherry	UNC Vector Core	N/A
AAV-DIO-RG	UNC Vector Core	N/A
AAV-CamKII-GCaMP6s-WPRE-SV40	Addgene	107790-AAV9
pAAV-Ef1a-Con/Foff 2.0-sRGECO	Addgene	137127-AAV8
pAAV-hSyn-Cre-P2A-dTomato	Addgene	107738-AAVrg
		
pAAV-hSyn-DIO-HA-hM3D(Gq)-IRES-mCitrine	Addgene	50454-AAV8
pAAV-CaMKIIa-HA-hM4D(Gi)-IRES-mCitrine	Addgene	50467-AAV8
**Chemicals, peptides, and recombinant proteins**		
Pilocarpine hydrochloride	Sigma	Cat: P6503
Clozapine N-oxide (CNO)	Sigma	Cat: C0832
Cholera toxin B (CTB) 488	Thermo Fisher	C22841
CTB-555	Thermo Fisher	C34776
CTB-647	Thermo Fisher	C34778
CIM0216	Tocris	5521
Ononetin	Tocris	5143
Mefenamic acid	Sigma	92574
4-AP	Tocris	0940
TTX	Hellobio	HB1034
Chromium V2 Reagents	10X	Various
**Experimental models: organisms/strains**		
Mouse: C57BL/6J	Jackson Laboratory	Stock: #:000664
Vglut2-ires-Cre	B. Lowell, Harvard Medical School	Stock: #:016963
PV-IRES-Cre	N. Uchida, Harvard University	Stock: #:008069
VGAT-Cre	Jackson Laboratory	Stock: 028862
**Recombinant DNA**		
Probe: Necab1	ACDBio	#428541-C2
Probe: Scn3a	ACDBio	#502641
Probe: Trpm3	ACDBio	#459911
Probe: Fos	ACDBio	#506921-C2, 506931-C2
**Software and algorithms**		
Adobe Creative Cloud	Adobe Inc.	5.9.0.373
ImageJ	National Institutes of Health	1.53v 21
MATLAB	MathWorks	N/A
R	R Core Team	R 4.2.0
Zeiss Zen	Carl Zeiss AG	N/A
IDPS	Inscopix/Bruker	1.6.0.3225
nVoke and nVue	Inscopix/Bruker	N/A
CellProflier	Broad Institute	4.2.1
LabView	National Instruments	N/A
pClamp	Molecular Devices	10.3
Axograph	John Clements	1.7.6

## Bullet points

- Social rank is encoded by plasticity in a forebrain-thalamocortical circuit
- High-rank mice show potentiated MDT→cACC inhibition during hierarchy establishment
- TRPM3 in MDT promotes neural excitability and enhanced competitive performance
- Cortical and basal forebrain inputs to MDT are differentially regulated by social rank
