# Supplementary material for: Molecular and neural control of social hierarchy by forebrain-thalamocortical circuit
Source: Cell. Author manuscript; Available in PMC 2025 Sep 25. (PMC12458795; doi:10.1016/j.cell.2025.07.024)
Supplement: 4 [file NIHMS2102803-supplement-4.pdf]

SOM Figure Legends

SOM Figure 1. Determinants of hierarchy formation among groups of unfamiliar mice. Related to Figure 1.

(A-D) Social hierarchy formation within groups of three males. **(A)** Paradigm for the emergence of social hierarchy among unfamiliar mice. SH: single housed; TT: tube test; RI: resident-intruder. **(B)** Elo ratings and ordinal ranks of an example group of three males. **(C)** Tube test hierarchy stability (left) and pairwise consistency (right) of observed groups compared to random outcomes; box plots show median and first and third quartiles. **(D)** Standard deviation in delta Elo scores by tube test rank (N = 10 groups of 3 mice, effect. Horizontal line denotes mean standard deviation. Per-rank mean ± s.e.m. are shown as dots on to the right of the raw data; error bars not overlapping the horizontal mean line are significant. **(E)** Duration of defensive behavior by rank. **(F)** Defensive index by rank (effect of rank: $p<0.05$). **(G)** R-squared ± 95% c.i. represents variance in tube rank explained by defensive index and duration (relative duration index = 0.22, absolute duration = 0.02). **(H)** Probability of attacking behavior by high and low ranks. **(I)** Urine swabbing experiment transferring saliva, urine or both from rank-1 to rank-4 and vice versa. In a majority of trials, rank-4 males gained higher rank when swabbed with rank-1 urine. N = 4 social groups (16 mice). **(J)** Example Elo (left) and delta Elo (right) from a group of four when rank-4 was swabbed with urine from rank-1 (shaded rectangles). **(K)** Ultrasonic vocalizations were emitted during resident-intruder (bottom) but not tube test (top). N = 2 groups (8 mice). Binomial logistic regression: panel E. *** $p<0.001$. Data is plotted as mean ± s.e.m.

SOM Figure 2. Social rank-dependent brain activity and the requirement of MDT in emergence of social hierarchy. Related to Figure 2.

(A) Quantification of brain-wide *Fos* expression in rank-1, rank-4, and control males. Mean ± s.e.m. LMER with Tukey post hoc test. N = 4 social groups plus two control males (10 mice). Equivalent to Fig. 2A. **(B)** Correlation matrices of *Fos* expression across brain regions in rank-1

and rank-4 mice (left and middle), and their difference (right), N = 4 social groups. **(C)** Ratio of *Fos*-positive to *Vglut2*-positive neurons (yellow) and ratio of *Vglut2*-positive to *Fos*-positive neurons (gray) in the MDT following tube test interactions. N = 11,849 *Vglut2*-positive cells (2 mice). (**D**) Frequency of aggressive behavior during resident-intruder interactions among the naïve cohorts. **(E-H)** Effects of MDT lesion on hierarchy dynamics. Mean hierarchy stability and consistency during tube test tournaments in naïve (**E**) and experienced (**F**) groups compared to sham controls. Tube test decision timing in naïve **(G)** and experienced **H)** groups compared to sham controls; dotted lines denote the timing of lesion and fitted lines are the predicted values of the linear model. Mean ± s.e.m, N = 8 social groups. **(I-L)** Effect of MDT lesion on pheromonal signaling and performance (change in Elo). In swab experiments, all mice were washed and rank-4 mice swabbed with rank-1 urine in sham **(I,J)** and lesion **(K,L)** groups, LMER with Tukey post hoc test, mean ± s.e.m, N = 6 social groups (24 mice), **p<0.01, NS = not significant. **(M-N)** Effect of MDT lesion vs. sham controls on sociability **(M)** and social memory **(N)** on time spent in the three-chamber assay. Spaghetti plots connect same individuals across rooms of the three-chamber apparatus; violins show density and distribution of data, LMER, N = 6 social groups (24 mice). NS = not significant.

Abbreviations: AIv: agranular insular area, ventral. ENTl: entorhinal area, lateral. BLA: basal amygdaloid nucleus. cACC: caudal anterior cingulate. PIR: piriform cortex. OFC: orbitofrontal cortex. PL: prelimbic area. COA: cortical amygdalar area. IL: infralimbic cortex. MDT: mediodorsal thalamus. AUD: auditory cortex, dorsal area. MPOA: medial preoptic area. VMH: ventromedial hypothalamus. LS: lateral septum. MEA: medial nucleus of amygdala. CA2: cornu Ammonis 2 of the hhippocampus. CP: caudoputamen. PVT: paraventricular nucleus of the thalamus. ACB: nucleus accumbens. RSPV: retrosplenial cortex, ventral. NDB: diagonal band nucleus. PAG: periaqueductal grey. LH: lateral habenula. DMH: dorsomedial hypothalamus. MCC: midcingulate cortex.

SOM Figure 3. Functional role of cACC^{-PV} and MDT^{-Glut} neurons. Related to Figures 2 and 3.

(**A**) Effect of CNO alone on social rank in control animals (effect of rank: $p>0.05$). **(B-D)** Effect of inhibition of MDT^{Glut} on social rank. **(B)** Individual performance (change in Elo) in a hierarchy where the rank-1 male (yellow trace) received CNO (shaded rectangles). Gray lines represent the other three males. **(C)** Relationship between performance and final tube rank in untreated mice. **(D)** Effect of CNO in experiments where either rank-1 (left) or rank-4 (right) received CNO while the other three mice received vehicle. **(E)** UMAP distribution of excitatory marker genes. **(F)** UMAP representation of all molecular clusters and their identity. **(G-I)** Cellular heterogeneity of the MDT. **(G, left)** Dendrogram assembled by hierarchical clustering of average variable gene expression for each cluster shows inter-cluster relationships based on transcription. **(G, right)** MDT body (top) and ring (bottom) clusters. Shown are example marker genes and their UMAP distribution and expression in mouse brain. **(H)** *Necab1*-positive cells co-express *Fos* during tube test (RNAscope FISH). **(I)** *Necab1*-positive cells co-express *Fos* in both rank-1 and rank-3 mice. **(J)** Candidate genes differentially expressed between ranks in MDT ring cluster cells**.** Table shows genes at the intersection of gene set enrichment analysis (GSEA) and differential gene expression analysis. Signal to noise ratio (S2N) uses the difference of mean expression level (between rank-1 and rank-3), scaled by the standard deviation. The larger the signal-to-noise ratio, the larger the differences of the means. **(K)** GSEA: effect of social rank on the tyrosine kinase gene set. Tyrosine kinase genes (black vertical lines) are sorted with all other genes based on differential expression between rank-1 (left) and rank-3 (right). Enrichment score (red line) is a running sum (from left to right) that increases as tyrosine kinase genes are encountered and decreases when they are not. Genes left of the yellow line (peak enrichment score) are upregulated in rank-1; genes right of purple line are downregulated in rank-3. The correlation of gene expression with social rank is shown in the bottom histogram (green). P-values shown as

permutation test corrected for multiple testing (FDR). **(L)** Histogram of the number of *Trpm3* puncta per *Necab1*-positive cell, as determined by RNAscope image analysis. **(M-P)** Role of TRPM3 in MDT^{Glut} neuronal activity. (**M**) Examples of firing frequency vs. current curves of MDT neurons under bath application of ACSF (control, black) or TRPM3 antagonists (mefanamic acid, red and ononetin, light blue). (**N**) Summary of change in peak firing rate of MDT neurons in response to TRPM3 antagonists (N=5 neurons). **(O)** Voltage-clamp recording protocol. Top: Barium currents were recorded using a series of depolarizing test pulses (100 ms duration, from −110 to +30 mV, 5 mV increments) **with or without ononetin. Bottom:** Typical electrophysiological traces of inward barium currents in the presence of sodium and potassium blockers (left) and in the presence of ononetin (right)**. (P)** Peak barium (control) current according to rank. LMER: panel A. Wilcoxon rank-sum test: panel M. Data is plotted as mean ± s.e.m.

SOM Figure 4. Inputs to, and iIntrinsic properties of, MDT neurons. Related to Figure 4.

(A) Images of rabies injections into MDT of three separate animals. **(B)** Inset of MDT region shows co-localization of AAV-TVA-mCherry and RbV-GFP. **(C)** Location of recorded cells**.** Each dot represents a biocytin filled cell that was recorded, according to social rank and bregma coordinate. 60 neurons were recorded in current clamp, and 53 neurons were recorded in voltage clamp. Bottom: confocal images of representative biocytin-filled neurons colored (gold circles). (**D**) Experimental setup for activating optical actuators Chronos (with 465 nm light) and Chrimson (with 625 nm light). **(E)** Effect of 465nm and 625nm stimulation of OFC neuron transfected with AAV-CamKII-Chronos-GFP. **(F)** Examples of opsin injection sites in OFC (AAV-Chronos, left), BF (AAV-Chrimson, middle), and projections of basal forebrain in the MDT (right). **(G-I)** Excitatory inputs from BF to MDT. **(G)** Schematic depicting electrophysiological recordings of neurons in MDT slices in response to optogenetic activation of BF projections (AAV Chrimson). **(H)** Top: electrophysiological traces (grey: 5 trials, black: mean response of 10 consecutive trials) for

EPSCs in control animal in response to activation of basal forebrain projections (light pulses: 5 ms duration, 625 nm at 5 Hz). Bottom: same paradigm for rank-3 animals (mean response in purple). **(I)** Summary plots of paired pulse results in all ranks and control animals. **(J-K)** Chemogenetic manipulation of OFC to MDT projections. **(J)** Example of OFC DREADD injection and cannula implant location in MDT. **(K)** Summary schematic of effects of chemogenetic manipulations of OFC→MDT projections with inhibitory and excitatory DREADDs.

SOM Figure 5. MDT projections to the ACC: functional role regulation of ACC excitatory/inhibitory balance. Related to Figure 5.

(A-C) Monosynaptic retrograde tracing from $cACC^{PV}$ neurons. **(A)** Retrograde tracing scheme. **(B)** Overview of cACC and labeling of rabies virus (green), helper AAVs (red), and starter neurons (white arrows) (left). Rabies-positive input neurons in the MDT (right). **(C)** Input fractions to $cACC^{PV}$ neurons (percent of total counted inputs), N = 3 mice. **(D - E)** Experimental setup for chemogenetic excitation of MDT→cACC pathway while inhibiting the dmPFC. **(F)** Effect of CNO on competitive performance in experiments where either rank-1, rank-2, or rank-3 received CNO while the other mice received vehicle. N = 2 groups; N = 6 mice. **(G)** MDT fibers labelled with Chronos (AAV-hSyn-Chronos-GFP) in cACC; $cACC^{PV}$ neurons are shown in red**.** **(H)** Schematic depicts the slice recording setup for evaluating the relationship between MDT^{cACC} firing and excitation-inhibition balance in cACC. **(I)** Example of simulated white noise and alpha function (left). Result of convolution of noise with alpha function and evoked spiking pattern in a ACC^{pyr} cell in response to injection of the noise (right).

SOM Figure 6. Timing of $cACC^{Pyr}$ and $dmPFC^{Pyr}$ activity. Related to Figure 6.

(A) Timing of activity in $cACC^{Pyr}$, $cACC^{PV}$, $dmPFC^{Pyr}$ and $dmPFC^{PV}$ during the tube test interaction of rank-1 vs rank-3. The dotted line depicts the time of meeting in the middle. **(B-C)** Timing of deviation in $cACC^{Pyr}$ and $dmPFC^{Pyr}$ baseline activity relative to the time of meeting in the middle

of the tube for **(B)** rank-1 vs. rank-2 and **(C)** rank-2 vs. rank-3. **(D)** Waterfall plots showing the activity patterns $cACC^{Pyr}$ neurons in a rank-2 male losing to rank-1 (left side) and winning against rank-3 (right side). Neurons are subdivided into activated and inhibited clusters according to the algorithm shown (bottom). **(E)** Summary of (D) showing the proportion of neurons according to cluster-type and wins/losses.

<u>SOM Video legends</u>

Video S1. DREADD manipulation of MDT^{Glut} in a low-rank animal, related to Figure 2.

Video of repeated tube test interactions between a rank-4 animal (left) and a rank-1 animal. Trials are color-coded according to whether they are under control conditions (grey) or when CNO is delivered to the rank-4 male (blue). Video is played at 2x real time.

Video S2. Selective TRPM3 antagonism in MDT in a mid-rank animal, related to Figure 3.

Video of tube test interactions between a rank-2 animal and a rank-3 animal. Each mouse had bilaterally implanted cannulas in MDT. Top: tube test between rank-2 and rank-3 under control conditions. Bottom: tube test between the same animals after 500 nL of a TRPM3 antagonist (ononetin) was injected into the MDT of rank-2.

Video S3. Optogenetic (Chronos) activation of BF→MDT projections in a low-rank animal, related to Figure 4.

Video of tube test interactions between a rank-2 animal and a rank-3 animal. Mice were from a cohort of 3 animals where all animals received bilateral injections of conditional Chrimson in BF and non-conditional Chronos in OFC along with optic fiber implants in MDT for light delivery. Top: tube test between rank-2 and rank-3 under control conditions. Bottom: tube test between the

same animals while blue light (465 nm, 5ms, 5 Hz at 1 mW/mm^2) pulses were delivered to the MDT of rank-3 mouse.

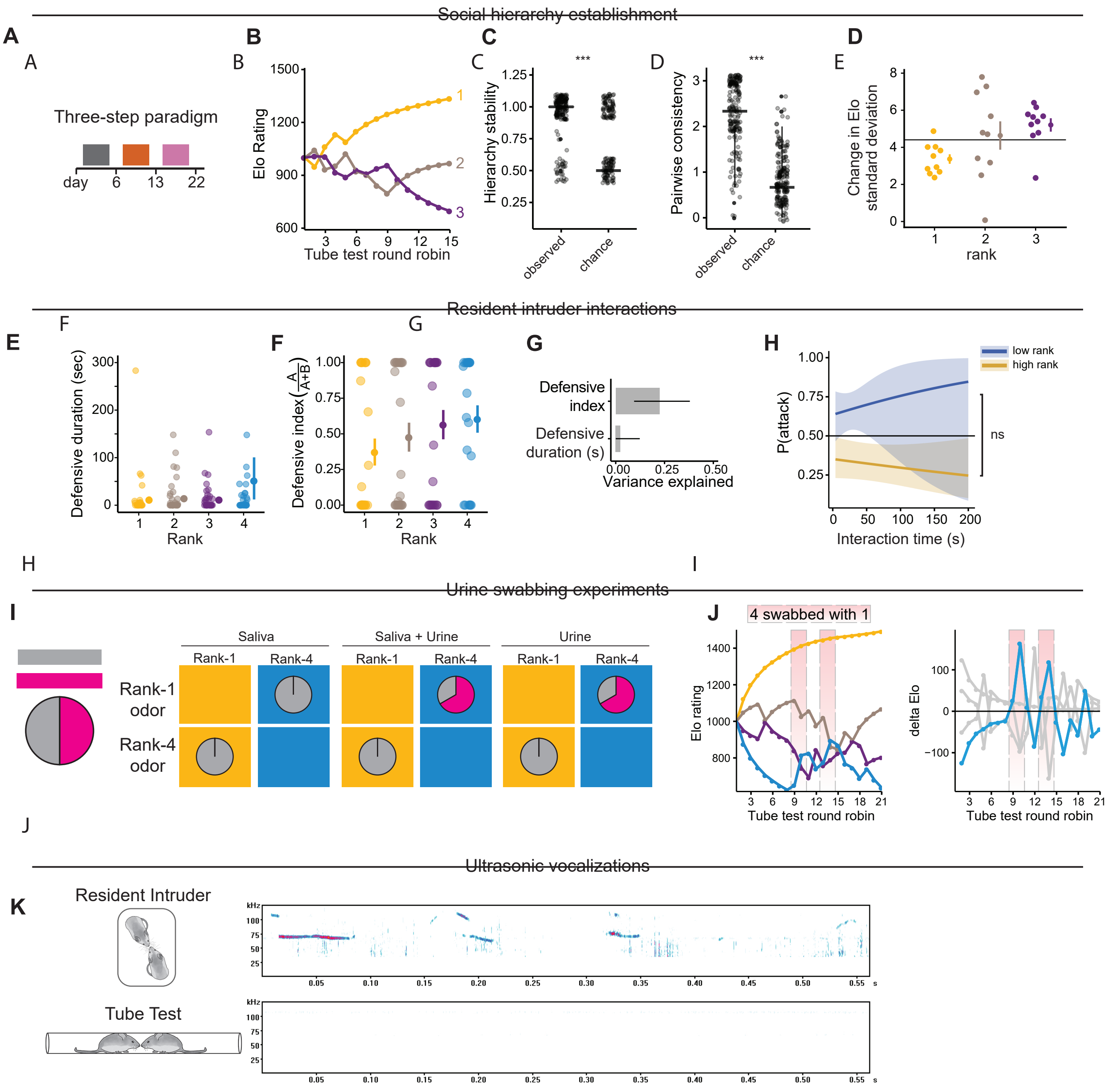

Social hierarchy establishment
A
B
C
D
E
Three-step paradigm
day
6
13
22
Elo Rating
Tube test round robin
1
2
3
Hierarchy stability
observed
chance
***
Pairwise consistency
Change in Elo standard deviation
rank
Resident intruder interactions
F
G
Defensive duration (sec)
Rank
Defensive index
Defensive index
Defensive duration (s)
Variance explained
H
P(attack)
low rank
high rank
ns
Interaction time (s)
I
Urine swabbing experiments
J
Saliva
Saliva + Urine
Urine
Rank-1
Rank-4
Rank-1 odor
Rank-4 odor
4 swabbed with 1
Elo rating
delta Elo
Tube test round robin
Ultrasonic vocalizations
K
Resident Intruder
Tube Test
kHz

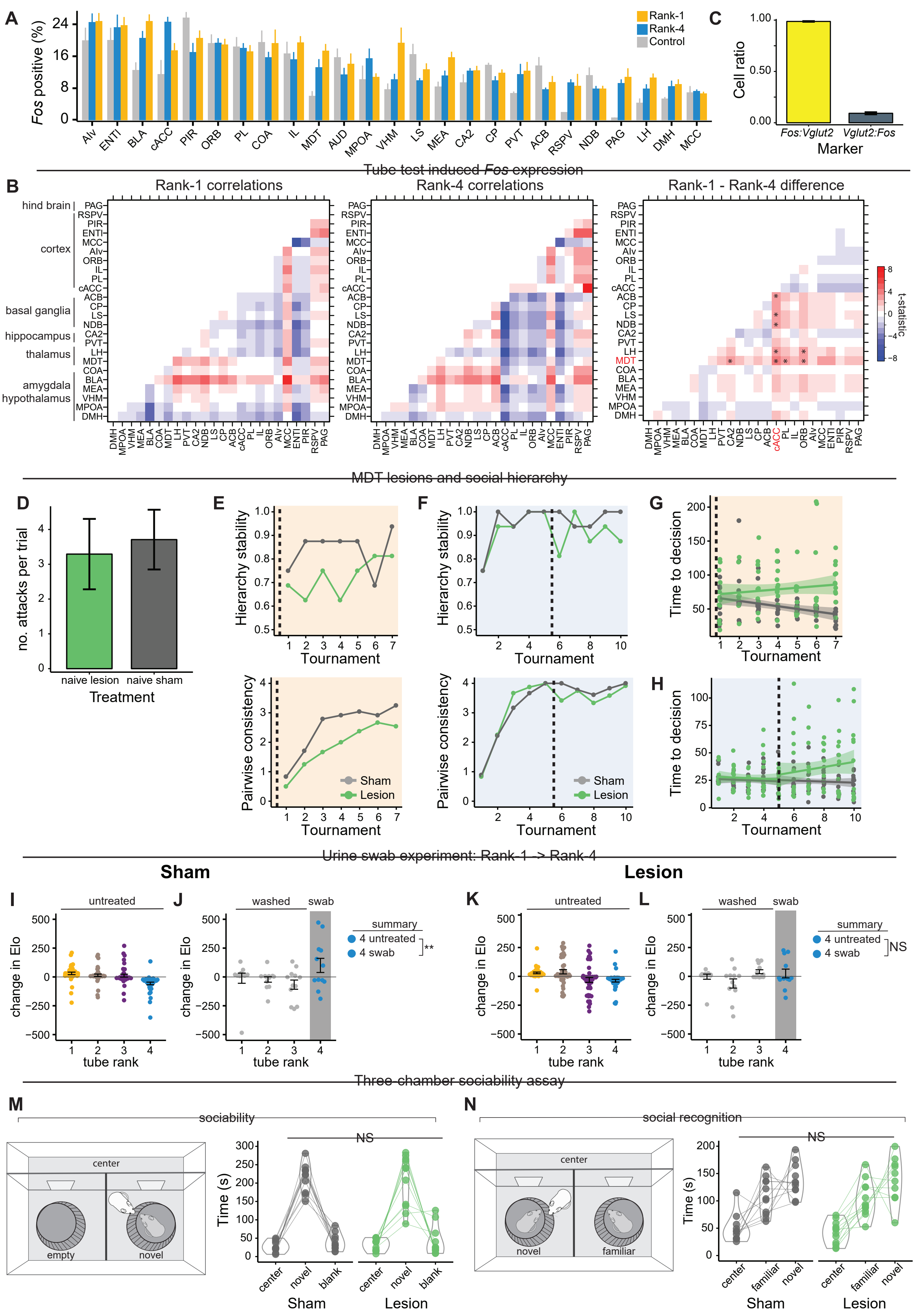
A
Fos positive (%)
Rank-1
Rank-4
Control
AIv ENTI BLA cACC PIR ORB PL COA IL MDT AUD MPOA VHM LS MEA CA2 CP PVT ACB RSPV NDB PAG LH DMH MCC
C
Cell ratio
Fos:Vglut2
Vglut2:Fos
Marker
Tube test induced Fos expression
B
Rank-1 correlations
Rank-4 correlations
Rank-1 - Rank-4 difference
hind brain
cortex
basal ganglia
hippocampus
thalamus
amygdala
hypothalamus
PAG RSPV PIR ENTI MCC AIv ORB IL PL cACC ACB CP LS NDB CA2 PVT LH MDT COA BLA MEA VHM MPOA DMH
t-statistic
MDT lesions and social hierarchy
D
no. attacks per trial
naive lesion
naive sham
Treatment
E
Hierarchy stability
Tournament
Pairwise consistency
Sham
Lesion
F
G
Time to decision
H
Urine swab experiment: Rank 1 -> Rank 4
Sham
Lesion
I
untreated
change in Elo
tube rank
J
washed
swab
summary
4 untreated
4 swab
**
K
L
NS
Three-chamber sociability assay
M
sociability
center
empty
novel
Time (s)
blank
N
social recognition
familiar

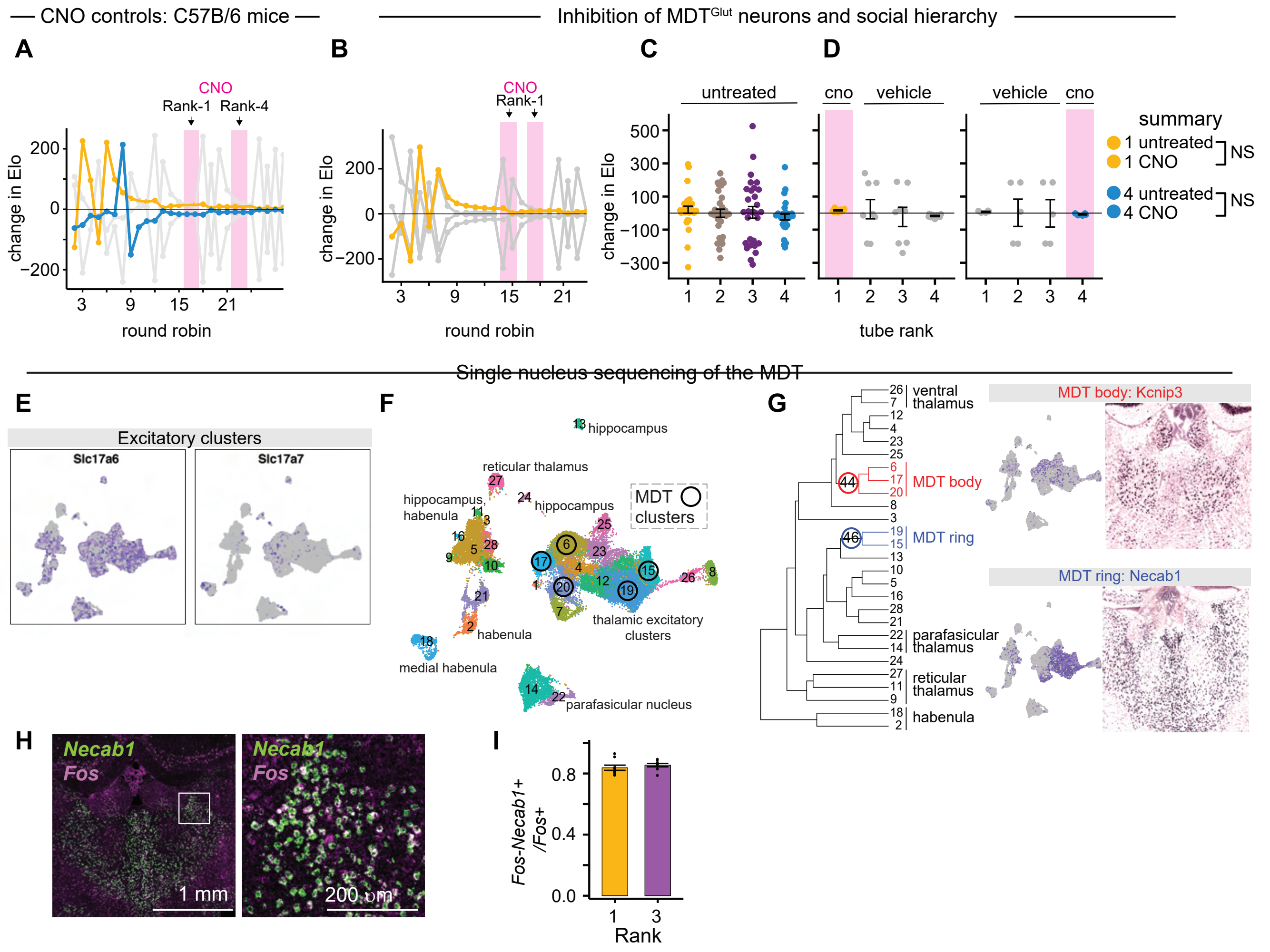

J Intersection of GSEA and differential gene expression analysis

GENE	S2N	Enrich. score	Gene Set	P value	log fold change	FDR
Mcoln2	0.16	0.04	vgic	1.9443E-06	0.125	0.0007
Dach1	0.16	0.06	repressor RNA polII	1.1801E-05	0.188	0.0030
Dctn4	0.13	0.03	enzyme_Tyrosine kinase	6.0978E-05	0.168	0.0096
Nfat5	0.09	0.01	activator RNA polII	0.00052008	0.159	0.0426
Trpm3	0.09	0.11	vgic	0.00213304	0.217	0.0895
Kcnh8	0.08	0.12	vgic	0.00446213	0.133	0.1277
Scn3a	0.10	0.06	vgic	0.00458675	0.109	0.1295
Cacna1b	0.09	0.09	vgic	0.00740269	0.123	0.1563
Tnk2	0.08	0.10	enzyme_Tyrosine kinase	0.00739803	0.120	0.1563
Borcs5	0.09	0.08	enzyme_Tyrosine kinase	0.00837638	0.119	0.1642
Dnah7c	0.09	0.06	enzyme_Tyrosine kinase	0.02194952	0.113	0.2250
Actr10	0.08	0.11	enzyme_Tyrosine kinase	0.04701106	0.114	0.2800
Kif2a	0.07	0.15	enzyme_Tyrosine kinase	0.07688226	0.115	0.3272
Dync1li2	0.07	0.19	enzyme_Tyrosine kinase	0.17438781	0.102	0.4139

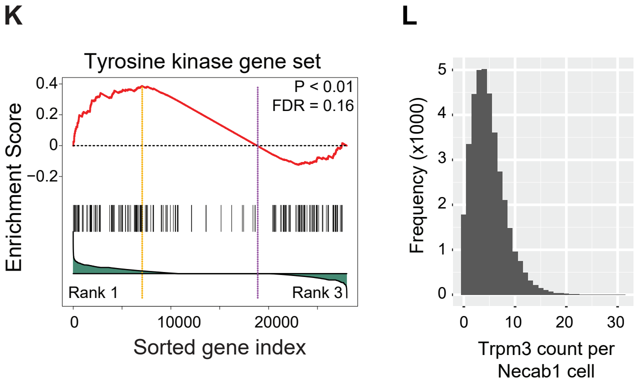

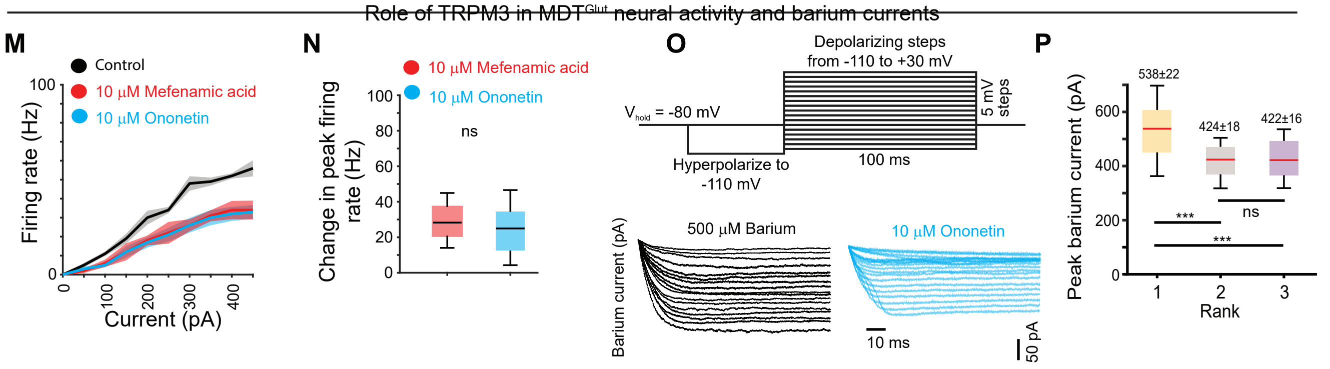

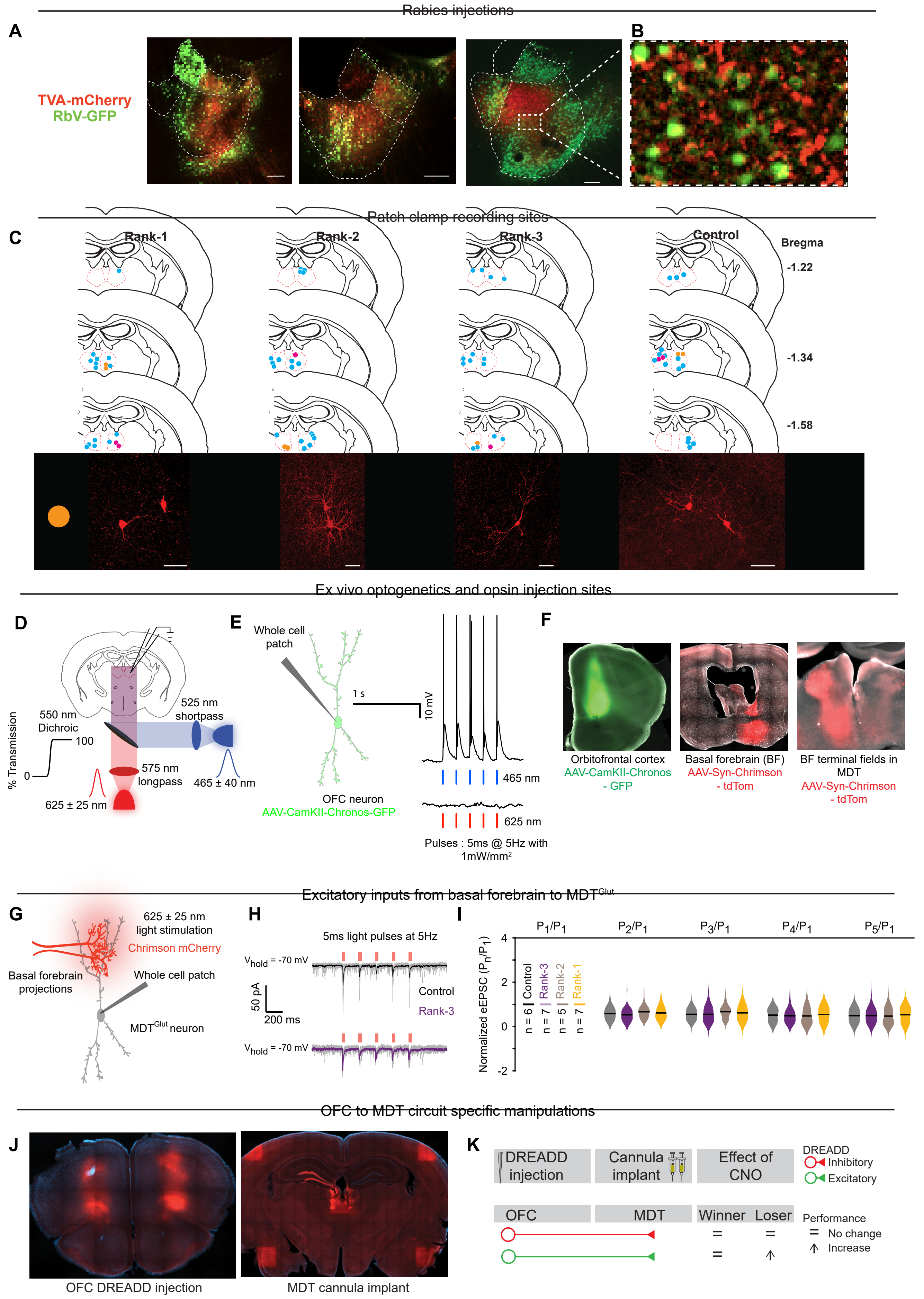

Rabies injections
A
B
TVA-mCherry
RbV-GFP
Patch clamp recording sites
C
Rank-1
Rank-2
Rank-3
Control
Bregma
-1.22
-1.34
-1.58
Ex vivo optogenetics and opsin injection sites
D
E
F
550 nm Dichroic
100
0
% Transmission
525 nm shortpass
575 nm longpass
625 ± 25 nm
465 ± 40 nm
Whole cell patch
1 s
10 mV
465 nm
625 nm
OFC neuron
AAV-CamKII-Chronos-GFP
Pulses : 5ms @ 5Hz with 1mW/mm²
Orbitofrontal cortex
AAV-CamKII-Chronos - GFP
Basal forebrain (BF)
AAV-Syn-Chrimson - tdTom
BF terminal fields in MDT
AAV-Syn-Chrimson - tdTom
Excitatory inputs from basal forebrain to MDT^Glut
G
H
I
625 ± 25 nm light stimulation
Chrimson mCherry
Basal forebrain projections
Whole cell patch
MDT^Glut neuron
5ms light pulses at 5Hz
V_hold = -70 mV
50 pA
200 ms
Control
Rank-3
V_hold = -70 mV
P1/P1
P2/P1
P3/P1
P4/P1
P5/P1
Normalized eEPSC (Pn/P1)
4
2
0
-2
n = 6 Control
n = 7 Rank-3
n = 5 Rank-2
n = 7 Rank-1
OFC to MDT circuit specific manipulations
J
K
OFC DREADD injection
MDT cannula implant
DREADD injection
Cannula implant
Effect of CNO
DREADD
Inhibitory
Excitatory
OFC
MDT
Winner
Loser
Performance
No change
Increase

MDT-ACC^{PV} connectivity

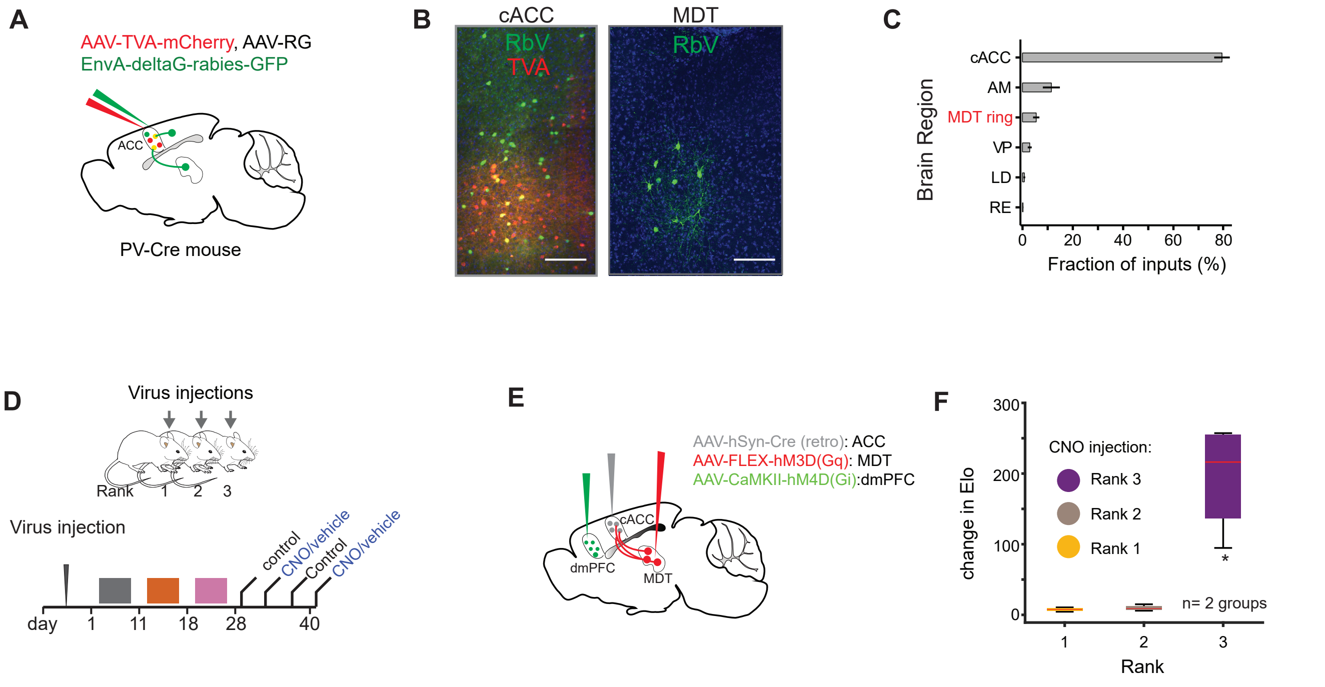

MDT-ACC^{PV} histology

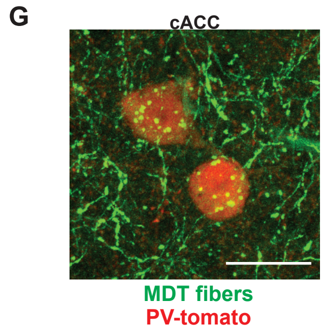

White noise whole cell patch clamp technqiue

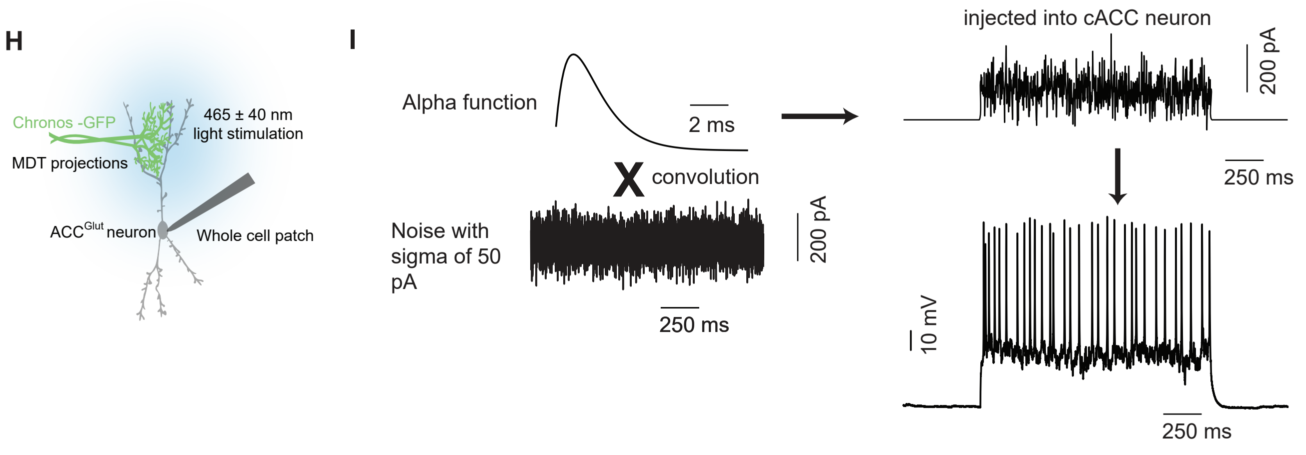

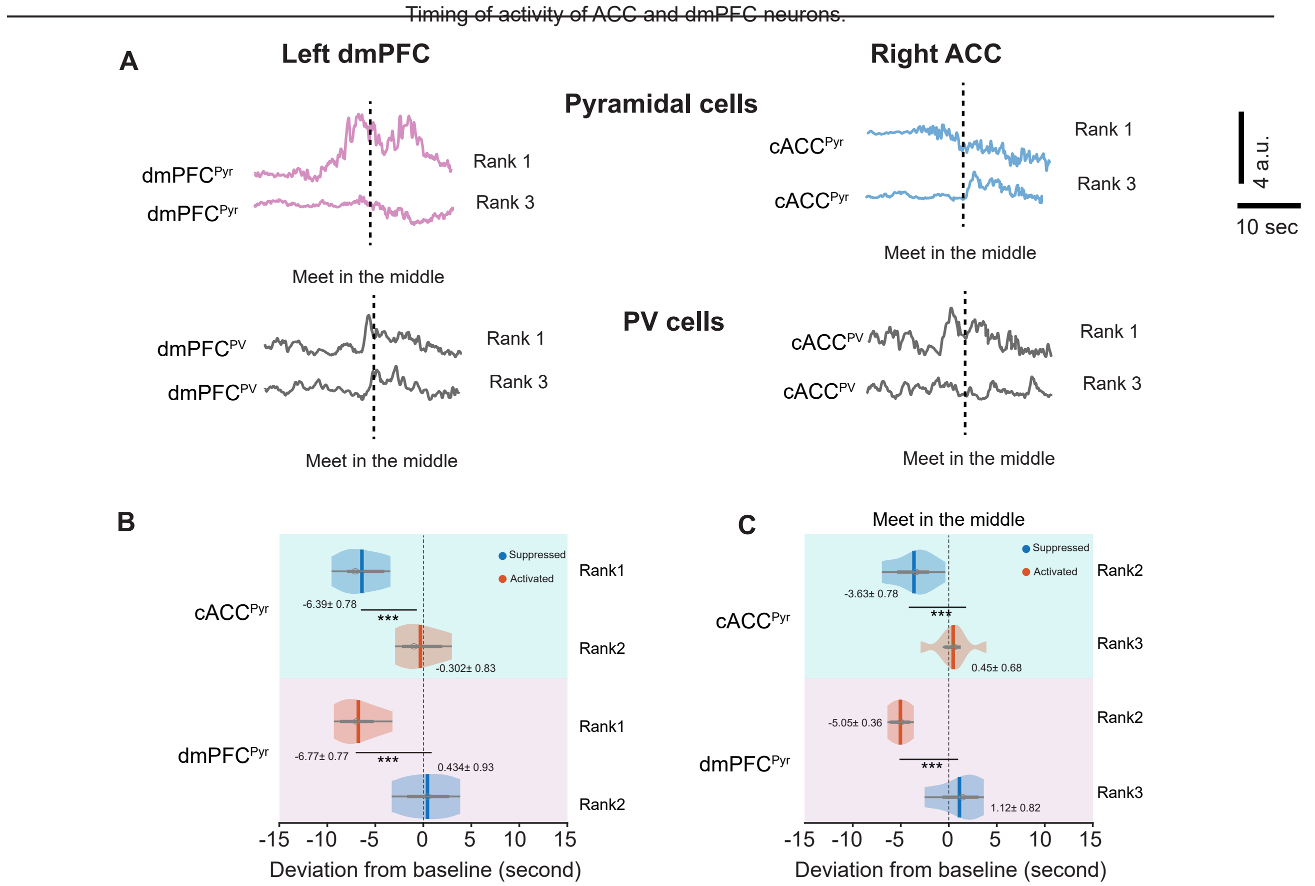

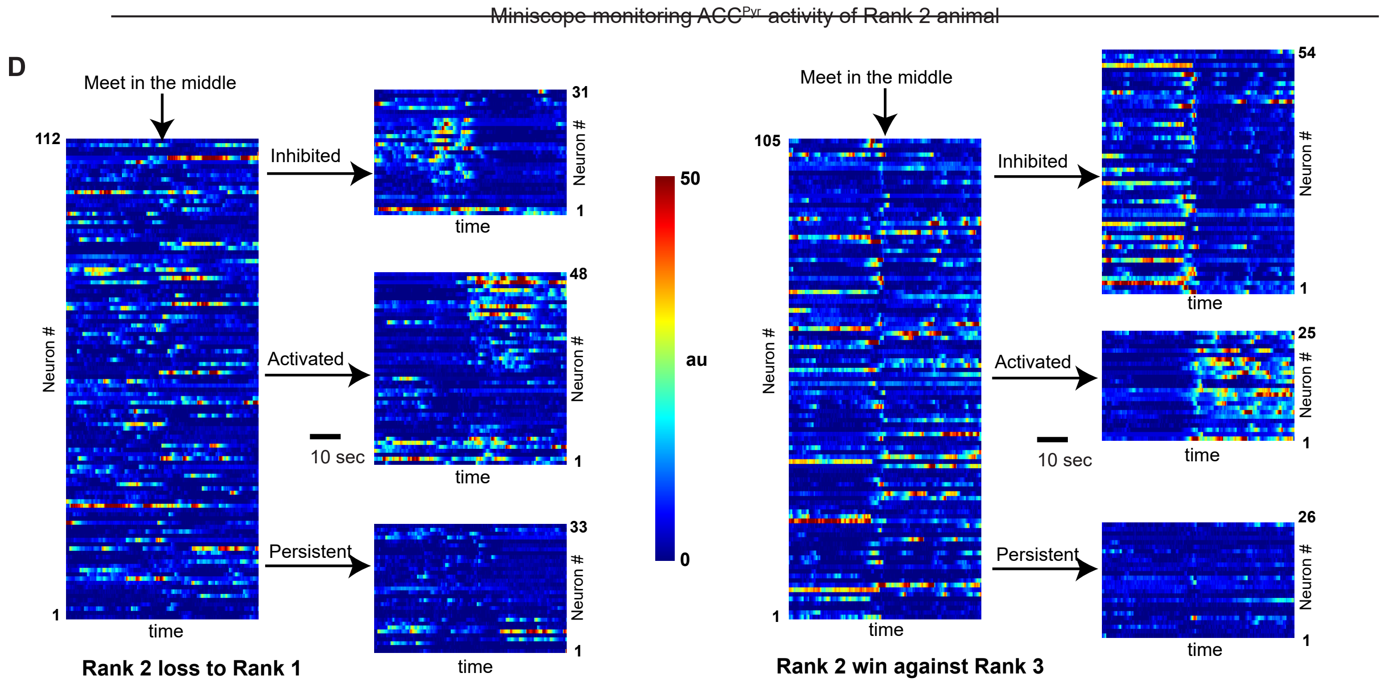

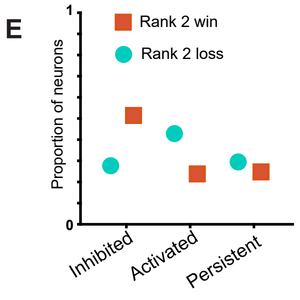

Activation: if ∑(activity 20 sec after meeting)-∑(activity 20 sec preceeding meeting) >= 2*std (activity over 60 sec)

Inhibition: if ∑(activity 20 sec preceeding meeting)-∑(activity 20 sec after meeting) >= 2*std (activity over 60 sec)
